# *In silico* molecular docking and *in vivo* evidence of whey protein concentrate-mediated protection against thioacetamide-induced intestinal toxicity in male albino rats via modulation of oxidative stress, inflammation, apoptosis, and fibrosis

**DOI:** 10.3389/fnut.2026.1817773

**Published:** 2026-05-13

**Authors:** Huda Fayez Al-Rashedi, Reem Alenazi, Nashmiah S. Alshammari, Mona A. Ibrahim, Sherif R. Mohamed, Hanan A. Okail

**Affiliations:** 1 Department of Biology, College of Sciences, University of Ha'il, Ha'il, Saudi Arabia; 2 Department of Zoology and Entomology, Faculty of Science, Capital University, Cairo, Egypt; 3 Department of Zoology, Faculty of Science, Sohag University, Sohag, Egypt

**Keywords:** thioacetamide, intestine, whey proteins, docking analysis, oxidative stress

## Abstract

Oxidative stress is a foremost cause in the etiology and progression of numerous illnesses. Thioacetamide (TA) has been demonstrated to promote oxidative stress and has been shown to be harmful in a variety of organs. The antioxidant activity of whey protein concentrate (WPC) has generated a lot of interest since it can help with the nutritional treatment of chronic disorders. The present study examined WPC’s antioxidant qualities, evaluating any potential defenses against TA-induced intestinal damage in rats. Forty rats were equally divided into four groups and treated for 5 days per week over 3 weeks: untreated control; TA (thioacetamide 100 mg/kg/day i.p.); WPC (whey protein 300 mg/kg/day oral); and WPC + TA (WPC orally followed 2 h later by TA i.p.). The results showed that TA treatment dramatically increased levels of nitric oxide and malondialdehyde while significantly decreasing levels of glutathione and activity of antioxidant enzymes (superoxide dismutase plus catalase) in intestinal tissue. Furthermore, TA injection showed higher values in apoptotic markers (Bcl-2 and Bax) and inflammatory indicators (IL-1*β* and TNF-*α*), and reduced expression of genes such as ZO-1 and HO-1 in intestinal tissue. Using a molecular docking study, the potential binding mechanisms of the antioxidant peptide with TGF-*β* and Keap1were examined. Additionally, there were notable immunopositive reactions for NF-kB and *α*. SMA, as well as significant histological changes, increased collagen fiber deposition, and duodenal goblet cell hyperplasia were observed in the TA group. However, WPC pretreatment significantly decreased intestinal tissue’s oxidative stress, pro-inflammatory, apoptotic, and fibrotic indicators, hence reducing TA-induced intestinal damage, suggesting WPC can be a useful and economical feed option that improves intestinal health.

## Introduction

Oxidative stress can cause the genesis and progression of many diseases, such as gastrointestinal diseases, and particularly promotes gut dysfunction. The mammalian intestine, a tightly controlled organ, is responsible for several vital processes, such as the absorption and digestion of nutrients (1). It also functions as a protective and a selectively permeable intestinal barrier through the intestinal epithelium, the primary cellular component of the intestinal barrier (2). However, toxins, infections, and other undesirable luminal antigens are prevented from paracellularly passing through the epithelium by tight junction protein components that seal the gaps between the epithelial cells (3). Cellular survival of the intestinal epithelium during oxidative stress is essential for maintaining body health, where damaged intestinal epithelial integrity may negatively impact other organs (4). The intestine’s complicated physiological and chemical milieu makes it vulnerable to a variety of stress injuries, which can result in inflammatory disorders like ulcerative or indeterminate colitis (5). Furthermore, oxidative stress harms the intestinal barrier by activating the immune system, causing inflammation (6), and decreasing tight junction protein expression, consequently causing increased permeability (7). Increased permeability can lower growth performance, cause diarrhea, and impair the immune system (8). Thioacetamide (TA), one of the most hepatotoxic compounds, is widely known for increasing oxidative stress and primarily affects tissues by overproduction of reactive oxygen species (ROS) (9, 10). TA’s overproduction of ROS causes DNA mutations and mitochondrial damage, which ultimately can induce fibrosis (11) and apoptosis (12, 13).

Although TA is classically recognized as a hepatotoxic agent, accumulating evidence indicates that TA induces oxidative stress and tissue damage in multiple extrahepatic organs, including the intestine, kidney, heart, and brain (9, 10), contingent upon the amount, duration, and environmental circumstances (10). Several studies have specifically demonstrated that TA administration induces significant biochemical and histological alterations in the rat small intestine, including diminished mucosal weight, reduced brush-border enzyme activities (alkaline phosphatase, ATPase, maltase, lactase), villus blunting, increased goblet cell count, lymphocyte infiltration, and ultrastructural changes in enterocytes (14, 15). Furthermore, TA has been shown to cause intestinal oxidative damage, increased bacterial translocation, and elevated plasma endotoxin levels (16).

The pathophysiological basis for TA-induced intestinal toxicity involves the generation of reactive oxygen species (ROS) following hepatic metabolism of TA to its reactive metabolite, TA-S-oxide, which enters the systemic circulation and reaches the intestinal mucosa (17). Once in the intestinal tissue, ROS overproduction triggers lipid peroxidation, depletion of antioxidant defenses, activation of inflammatory cascades, and disruption of tight junction proteins, ultimately compromising intestinal barrier integrity (6, 7). Importantly, the observed intestinal damage may result from both direct cytotoxic effects of circulating TA metabolites on intestinal epithelial cells and indirect effects secondary to liver injury, including systemic inflammation, portal hypertension, and altered gut-liver axis communication (18, 19). Studies have shown that TA-induced acute liver failure leads to gastrointestinal dysfunction, impaired intestinal motility, and intestinal wall damage, supporting the interplay between hepatic and intestinal injury (20).

Certain dietary components can act as immunoregulators, antioxidants, gene expression regulators, and modulators of many cellular processes, among other biological roles. However, there has been interest in the regulation of intestinal health through nutrition products that can act as natural antioxidants to maintain ROS equilibrium and, hence, adequate barrier function (21). Whey protein (WP), one of the most vital ingredients in dairy-based products, has grown in popularity because of its possible ability to reduce body weight (22). WP is typically taken out of the liquid part of milk when making cheese (23). WP differs in terms of protein concentration and processing level as a WP isolates (WPI), concentrates (WPC), and hydrolysates (WPH) (24). WPC, a protein-rich powder, is widely used in the production of infant and young child foods. Additionally, WPC has been shown to improve intestinal barrier function in human intestinal cells cultured *in vitro* (Caco-2) (25). WPC proteins include glycomacropeptides *β*-lactoglobulin, immunoglobulins, *β*-lactalbumin, and minor proteins, all with beneficial properties (22). These bioactive peptides work by chelating transition metal ions, preventing lipid peroxidation, and scavenging free radicals. WPC’s main active components are *β*-lactoglobulin and *α*-lactalbumin. *β*-lactoglobulin (50–55%) contains necessary branched-chain amino acids (26), which may bind to a variety of hydrophobic proteins, improving the absorption of lipid-soluble vitamins and other biologically active ingredients (27). *α*-lactalbumin (20–25%) may improve immunological function and reduce stress (28). Also, the antioxidant qualities of WPC are associated with its role in GSH synthesis. Sulfur-rich amino acids, especially cysteine, which is a precursor to GSH, are highly concentrated in WPC (29).

Nuclear factor erythroid2-related factor2 (Nrf2) is a significant reactive-sensitive transcriptional regulator that can help improve the body’s oxidative stress state, stimulate cellular survival, and maintain cell redox balance by activating and controlling the constitutive and inducible expression of antioxidant enzymes in cells (30). The Keap1-Nrf2 signaling pathway is a major regulator of the cell protection response, which is involved in exogenous and endogenous stress instigated by ROS (31). Nrf2 is at the center of this pathway regulation. The target gene’s regulatory region contains an antioxidant response element (ARE) that is first bound by Nrf2. Second, Nrf2 attaches itself to Keap1 in order to facilitate its breakdown via the ubiquitin-proteasome pathway (32). Additionally, NF-kB is an activated transcriptional regulator that controls a wide range of genes involved in inflammatory responses. NF-κB pathways can be triggered by numerous stimuli, including cytokine receptor ligands (33). Nrf2 and NF-κB pathways control cellular response to oxidative stress and inflammation through the underlying complex molecular mechanisms. By stimulating the Nrf2 pathway and preventing the NF-κB-mediated inflammation, the preventive candidates increase the antioxidant capacity and counteract organ damage. On the contrary, the protective agents enhance HO-1 expression to prevent NF-κB activation and either directly or indirectly stimulate Nrf2 signaling (32).

In an earlier study, feeding rats WPC showed a marked decrease in ethanol-induced lesions (34). In patients with intestinal bowel disease (IBD), protein nutritional supplementation can enhance the body’s nutritional status, lower inflammatory markers, lessen intestinal epithelium damage, and improve medical outcome indicators (35, 36). WP can boost antioxidative, anti-inflammatory, anti-apoptotic, and anti-fibrotic actions in the gut by activating the Keap1-Nrf2 signalling pathway. Its activation can boost the activity of Nrf2 associated antioxidant and detoxifying enzymes. Furthermore, the antioxidant-active peptides derived from WP, once digested *in vivo*, either bind to Keap1 or stop Keap1 and Nrf2 from binding. These peptides then raise the downstream gene HO-1’s transcription and expression, reducing oxidative stress and inflammation (37, 38). Also, WP inhibits NF-κB, a key regulator of intestinal inflammation, which contributes to its anti-inflammatory effect (39). According to research on animals, giving IBD rats a daily dose of whey or quinoa protein can dramatically lower the expression of NF-κB in their colon (40, 41).

A strong correlation exists between the increasing occurrence of intestinal illnesses and food habits (42). Given the direct interactions between the intestinal mucosa and food components, which necessitate the use of foods with high nutritional value. According to several studies that have demonstrated that WPC is important for improving the body’s nutritional condition, lowering inflammatory markers, and preventing intestinal mucosal damage, we hypothesized that whey protein would be a good choice for a safe and effective dietary approach to prevent oxidative cell damage, investigating in greater detail the additional mechanisms by which WPC nutritional supplementation reduces oxidative stress. Therefore, this study aimed to evaluate the protective effects of whey protein concentrate (WPC) against thioacetamide (TA)-induced intestinal toxicity in male albino rats. We hypothesized that WPC pretreatment would attenuate TA-induced intestinal damage through modulation of oxidative stress, inflammation, apoptosis, and fibrosis. The specific objectives were: (1) to assess the antioxidant capacity of WPC by measuring oxidative stress markers (MDA, NO) and antioxidant enzymes (GSH, SOD, CAT); (2) to evaluate inflammatory (IL-1*β*, TNF-*α*) and apoptotic (Bax, Bcl-2) markers; (3) to examine the expression of tight junction (ZO-1) and antioxidant (HO-1) genes; (4) to investigate histopathological and immunohistochemical changes (NF-κB, α-SMA, collagen deposition); and (5) to perform molecular docking analysis to predict the binding interactions of whey-derived bioactive components with KEAP1 and TGF-*β* receptors.

## Materials and methods

### Chemicals

Whey Protein Concentrate (WPC) 100% was acquired from Imtenan Health Shop in Cairo, Egypt, while Thioacetamide (TA) was sourced from Sigma-Aldrich Corp., St. Louis, MO, United States.

#### Characterization of whey protein concentrate

Whey Protein Concentrate (WPC) 100% natural was obtained from Imtenan Health Shop (Cairo, Egypt). According to the manufacturer’s product specifications, per 100 g of powder: protein 68 g, fat 0 g, carbohydrates not specified by manufacturer, and 68 calories. The product contains whey protein concentrate, milk protein concentrate, fat-free milk, maltodextrin, sucralose, salt, natural strawberry flavor, red beets powder, and silicon dioxide. No artificial colors or preservatives are added. The WPC was stored at 4 °C in a sealed container, and fresh solutions were prepared daily in distilled water for oral gavage.

### Experimental protocol

Forty adult male albino rats (180–200 g; 7–9 weeks old) were obtained from the animal unit at Sohag University, Egypt. The rats were allowed unlimited access to food and water during their one-week acclimatization period in the lab. This study followed the ethical guidelines of the Committee for Scientific Research Ethics (CSRE). The protocol number (SU-FS-10-26) was approved by the Sohag University Faculty of Science Institutional Animal Care and Use Committee (SU-FS-IACUC).

Upon completion of acclimatization, the rats were split into four equal groups (*n*=10) by random selection. The group in control: In this group, rats were given intraperitoneal injections of normal saline (NaCl 0.9%) and oral distilled water. The second group. TA dissolved in 0.9% NaCl was given intraperitoneally (100 mg/kg/day) (43) to induce intestinal toxicity in the rat model. Significant intestinal damage coincided with severe liver damage in the TA toxicity (19). According to Rungratanawanich et al. (18), oxidative stress induces post-translational protein changes via cytochrome P450-2E1, the principal TA metabolizer, resulting in immunological activation, cellular malfunction, and, eventually, cell death and damage in numerous organs. Then the oxidative proteins and gut damage/leakiness happen rapidly after drug exposure, set off a series of molecular alterations that eventually lead to liver injury, which is often seen later. Also, liver damage were all visible two and four weeks following TA exposure. Pathological alterations throughout time indicate that gut leakiness develops prior to liver fibrosis (44).

In the third group, rats received a daily oral dose of 300 mg/kg of WPC, this dose was selected based on previous rat studies demonstrating antioxidant and anti-inflammatory effects without toxicity (45). Converting to human equivalent dose using body surface area (rat to human: divide by 6.2) gives approximately 48 mg/kg/day for a 60 kg human, or ≈2.9 g/day (46). This is within the range of typical whey protein supplementation (20–40 g/day) when adjusted for protein content (68% protein in our WPC, giving ≈1.97 g pure protein/day), suggesting translational relevance. However, direct clinical extrapolation requires caution due to differences in gut physiology, metabolism, and disease etiology. The fourth group of rats received an oral dose of 300 mg/kg WPC, and then, after 2 h, were injected with 100 mg/kg of TA. All treatments were administered for 3 weeks, 5 days a week. Rats were deeply anesthetized using thiopental sodium (20 mg/kg, intraperitoneal injection), followed by euthanasia via cervical dislocation under deep anesthetized. Following dissection and cleaning, the duodenum was sectioned. One portion underwent histological processing; the other was homogenized in cold medium, centrifuged (3,000 rpm, 4 °C, 10 min), and the supernatant was retained for biochemical assays.

### Molecular docking study

The crystal structures of Kelch-like ECH-associated protein 1 (KEAP1; PDB ID: 4IQK) and transforming growth factor-*β* receptor I (TGF-*β* RI/ALK5; PDB ID: 1VJY) were retrieved from the Protein Data Bank (47). All water molecules and co-crystallized ligands were removed prior to docking, and polar hydrogen atoms and Kollman charges were added to prepare the receptors. Ligands representing whey protein-derived bioactive components, namely cysteine and glutathione, were obtained from the PubChem database and energy-minimized before docking. Sulforaphane was included as a positive control inhibitor for KEAP1.

Rationale for ligand selection: Cysteine and glutathione were selected because: (i) whey protein is rich in cysteine, a rate-limiting precursor for intracellular glutathione synthesis (48); (ii) glutathione is the principal endogenous antioxidant that directly neutralizes ROS and regenerates other antioxidants (49); and (iii) both molecules have been shown in previous studies to interact with KEAP1 and TGF-*β* signaling components. Sulforaphane, a well-characterized Nrf2 activator, was used as a positive control for KEAP1 binding (50). Molecular docking was performed targeting the Nrf2-binding site of KEAP1 and the ATP-binding pocket of ALK5 to evaluate the potential antioxidant and anti-fibrotic mechanisms. AutoDock Vina (version 1.2.6) was used to conduct molecular docking simulations, targeting the Nrf2-binding site of KEAP1 and the kinase domain of TGF-*β* Receptor I (ALK5) (51). Binding affinities and interaction patterns were analyzed directly from the docking results, focusing on hydrogen bonds, key residues, and binding energies to clarify possible molecular pathways underlying the protective influences of whey protein against thioacetamide (TA)-induced intestinal inflammation.

### Biochemical assays

Preparation of Intestinal Homogenates: Duodenal tissue samples were rinsed with ice-cold phosphate-buffered saline (PBS, pH 7.4) to remove blood and debris. Each sample was blotted dry, weighed, and minced with fine scissors. Tissue was homogenized in ice-cold lysis buffer containing 50 mM Tris–HCl (pH 7.4), 150 mM NaCl, 1 mM EDTA, 1% Triton X-100, and a protease inhibitor cocktail (Roche, Basel, Switzerland) at a ratio of 1:10 (w/v). Homogenization was performed using a glass-Teflon homogenizer (10 strokes, 4 °C). The homogenate was centrifuged at 3,000 rpm for 10 min at 4 °C. The resulting supernatant was aliquoted and stored at −80 °C until biochemical analysis.

Protein quantification: Total protein concentration in intestinal homogenates was determined using the Bradford method (Bio-Rad Protein Assay Kit, Cat# 500-0006, Hercules, CA, United States) with bovine serum albumin as standard (52). Absorbance was read at 595 nm. All biochemical parameters (MDA, NO, GSH, SOD, CAT) and ELISA results (IL-1*β*, TNF-*α*, Bax, Bcl-2) were normalized to protein content and expressed as per mg of protein.

### Analysis of oxidative and antioxidant markers

The manufacturer’s instructions and commercial kit procedures were followed for all evaluation tests and kits (Bio-Diagnostic Co., Giza, Egypt). Malondialdehyde (MDA) Assay: Oxidative stress in intestinal homogenates was assessed by quantifying malondialdehyde (MDA) using a colorimetric kit (Bio-Diagnostic, Cat# MD 25 29) based on the reaction of MDA with thiobarbituric acid (TBA) in an acidic medium at 95 °C to form a thiobarbituric acid reactive product (TBARS) with pink color measured at 534 nm, with results expressed as nmol/mg protein according to Ramos-Vara et al. (53). Nitric Oxide (NO) Assay: The level of nitric oxide (NO) was measured using a colorimetric kit (Bio-Diagnostic, Cat# NO 25 33) based on the Griess reaction, where NO is rapidly oxidized to nitrate (NO₃^−^) and nitrite (NO₂^−^), and nitrate is converted to nitrite using nitrate reductase, followed by reaction with Griess reagents to form a purple/reddish azo compound measured at 540 nm, with results expressed as nmol/mg protein following Archer’s technique (54). Glutathione (GSH) Assay: Glutathione (GSH) level was assessed using a colorimetric kit (Bio-Diagnostic, Cat# GS 25 17) based on the method of Beutler et al., where 5,5′-dithiobis (2-nitrobenzoic acid) (DTNB; Ellman’s reagent) reacts with reduced GSH to form a yellow compound measured at 405 nm, with results expressed as nmol/mg protein as outlined by Beutler et al. (55). Superoxide Dismutase (SOD) Assay: Superoxide dismutase (SOD) activity was assayed using a colorimetric kit (Bio-Diagnostic, Cat# SD 25 20) based on the inhibition of phenazine methosulphate (PMS)-mediated reduction of nitroblue tetrazolium (NBT) dye, with absorbance measured at 560 nm, with results expressed as U/mg protein according to Nishikimi et al. (56). Catalase (CAT) Assay: Catalase (CAT) activity was measured using a colorimetric kit (Bio-Diagnostic, Cat# CA 25 17) based on Aebi’s method. The assay determines CAT activity by measuring the reduction of hydrogen peroxide (H₂O₂) over 1 min. The reaction is stopped with a catalase inhibitor, and remaining H₂O₂ reacts with 3,5-dichloro-2-hydroxybenzene sulfonic acid and 4-aminophenazone in the presence of peroxidase to form a colored product measured at 510 nm. The color produced is inversely proportional to the amount of catalase in the sample. Results are expressed as U/mg protein. This method follows the original Aebi principle (57).

### Analysis of inflammatory plus apoptotic markers

Inflammatory markers (IL-1*β* and TNF-*α*) and apoptotic markers (Bax and Bcl-2) were quantified in intestinal tissue homogenates using commercial sandwich ELISA kits from Sunlong Biotech Co., Ltd. (Hangzhou, China). IL-1*β* was measured using a rat-specific ELISA kit (Sunlong Biotech, Cat# EL0040Ra; assay range 31.25–2000 pg./mL, sensitivity 1.42 pg./mL) with absorbance read at 450 nm. Results were expressed as pg./mL. TNF-*α* was measured using a rat-specific ELISA kit (Sunlong Biotech, Cat# EL0013Ra; assay range 4.69–300 pg./mL, sensitivity 0.43 pg./mL) with absorbance read at 450 nm. Results were expressed as pg./mL. Bax was measured using a rat-specific ELISA kit (Sunlong Biotech, Cat# SL0109Ra; assay range 10–800 pg./mL, sensitivity 2.5 pg./mL) with absorbance read at 450 nm. Results were expressed as pg./mL. Bcl-2 was measured using a rat-specific ELISA kit (Sunlong Biotech, Cat# SL0108Ra; assay range 18–1,000 pg./mL, sensitivity 4.5 pg./mL) with absorbance read at 450 nm. Results were expressed as pg./mL.

### Real-time PCR analysis

Quantitative real-time PCR analysis was performed to determine duodenal HO-1 and ZO-1 mRNA expression.

#### RNA extraction

Total RNA was extracted from blood samples using the Direct-zol RNA Miniprep Plus kit (Cat# R2072, ZYMO RESEARCH CORP., United States). This streamlined method, designed for samples preserved in TRI Reagent®, involved adding ethanol, binding RNA directly to a Zymo-Spin™ Column, performing on-column DNase I (Merck) digestion to eliminate genomic DNA, and eluting high-quality RNA. RNA quantity and quality were subsequently assessed using a Beckman dual spectrophotometer (USA).

#### Gene expression analysis

Gene expression was then analyzed using the SuperScript™ IV One-Step RT-PCR System with ROX components (Cat# 12594100, Thermo Fisher Scientific, Waltham, MA, United States). Each 50 μL reaction mixture contained 0.5 μL SuperScript™ IV RT Mix, 25 μL of 2X Platinum™ SuperFi™ RT-PCR Master Mix (which includes SYBR Green dye, ROX dye, thermostable M-MuLV reverse transcriptase, and Taq DNA polymerase), 2.5 μL each of forward and reverse primers (10 μM), 10 μL template RNA (5 ng/μL), and 9.5 μL nuclease-free water. The following primer sequences were used: rat ZO-1 (NM_001437494.1) sense, 5-CACACGATGCTCAGAGACGAAGG-3 and antisense, 5-CTGTATGGTGGCTGCTCAAGGTC-3; rat HO-1 (NM_012580.2) sense, 5-GAGCGCCCACAGCTCGACAG-3 and antisense, 5-GTGGGCCACCAGCAGCTCAG-3; and GAPDH (XM_032916238.1) sense, 5-AACGACCCCTTCATTGAC-3 and antisense, 5-TCCACGACATACTCAGCAC-3.

The prepared samples were loaded into a StepOne real-time PCR system (Applied Biosystems, Foster City, United States) and subjected to the following thermal cycling conditions: reverse transcription at 55 °C for 15 min, RT enzyme inactivation at 95 °C for 2 min, followed by 40 amplification cycles of denaturation at 95 °C for 10 s, annealing at 55 °C for 10 s, and extension at 72 °C for 30 s, with a final extension at 72 °C for 5 min. The high processivity of the enzyme enabled efficient cDNA synthesis, and the fluorescence of SYBR Green upon binding to double-stranded DNA permitted real-time detection. Relative gene expression (RQ) for each target was calculated using the 2^−∆∆Ct^ method, with values normalized to the GAPDH housekeeping gene and calibrated against a control sample (58).

### Histological and histochemical analysis

Histological examination of the duodenum involved formalin fixation, paraffin embedding, sectioning at 5 μm, and hematoxylin and eosin staining (59). Masson’s trichrome dye was used to distinguish collagen fibers on other slides, as previously reported (60). Duodenal tissue sections were also subjected to Alcian blue staining, which specifically highlights acid mucins contained within goblet cells.

### Immunohistochemical staining

Intestine tissue sections underwent immunohistochemistry using Anti-NF-κB p65 (ab16502) and *α*-smooth muscle actin (α-SMA) (ab21027) antibodies from Abcam, Cambridge, United Kingdom. Staining was executed according to the manufacturer’s guidelines, employing the DAB chromogenic substrate alongside the Expose Mouse and Rabbit Specific HRP/DAB Detection Kit (Abcam; catalog # ab80436).

### Morphometric analysis

The histologic injury score for the small intestine was evaluated according to a previously established five-point scale (61). This scoring system was applied uniformly to all intestinal segments, including the duodenum. Briefly, a score of 1 denoted no histologic damage, a score of 2 indicated epithelial cell lifting with the majority of villi intact, a score of 3 (the threshold for histologic NEC) represented necrosis extending to the mid-villus level with concomitant villus blunting, a score of 4 was assigned for complete necrosis of villi, with possible pneumatosis intestinalis, and a score of 5 designated the most severe injury, characterized by transmural necrosis, scant villi, and widespread pneumatosis. Duodenal tissue slides stained with H&E were examined in a blinded fashion by at least two independent graders, including a board-certified pediatric pathologist. To calculate the mean area percentage of collagen fibers, NF-κB, and *α*-SMA-positive immunoreactivity, ten high-power fields (40 × magnification) from each intestinal tissue segment. For each intestinal section, the total count of goblet cells was measured along the complete crypt-villus axis from ten properly aligned crypt-villus structures (62). Statistical analysis of the data was performed using ImageJ software (version 1.46, NIH, United States).

### Statistical analysis

All data were compiled and processed using GraphPad Prism® version 5 (GraphPad Software Inc., San Diego, California, United States). The results are presented as the mean accompanied by the standard deviation of the mean (mean±SD). Intergroup differences were evaluated employing a one-way analysis of variance (ANOVA). Subsequently, pairwise comparisons between groups were conducted using Tukey’s *post hoc* test. The threshold for statistical significance was established at a *p*-value less than or equal to 0.05.

## Results

### Molecular docking

**Figure 1:**
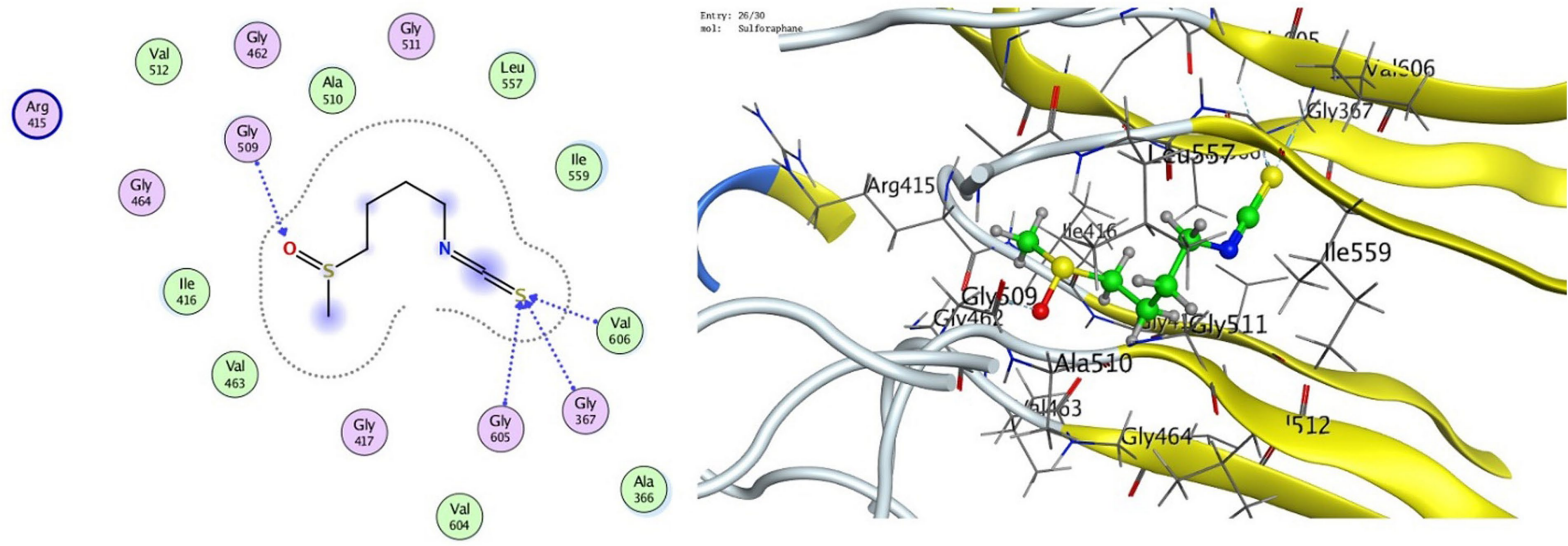
2D, 3D docking of sulforaphane (positive control) with KEAP1.

**Figure 2:**
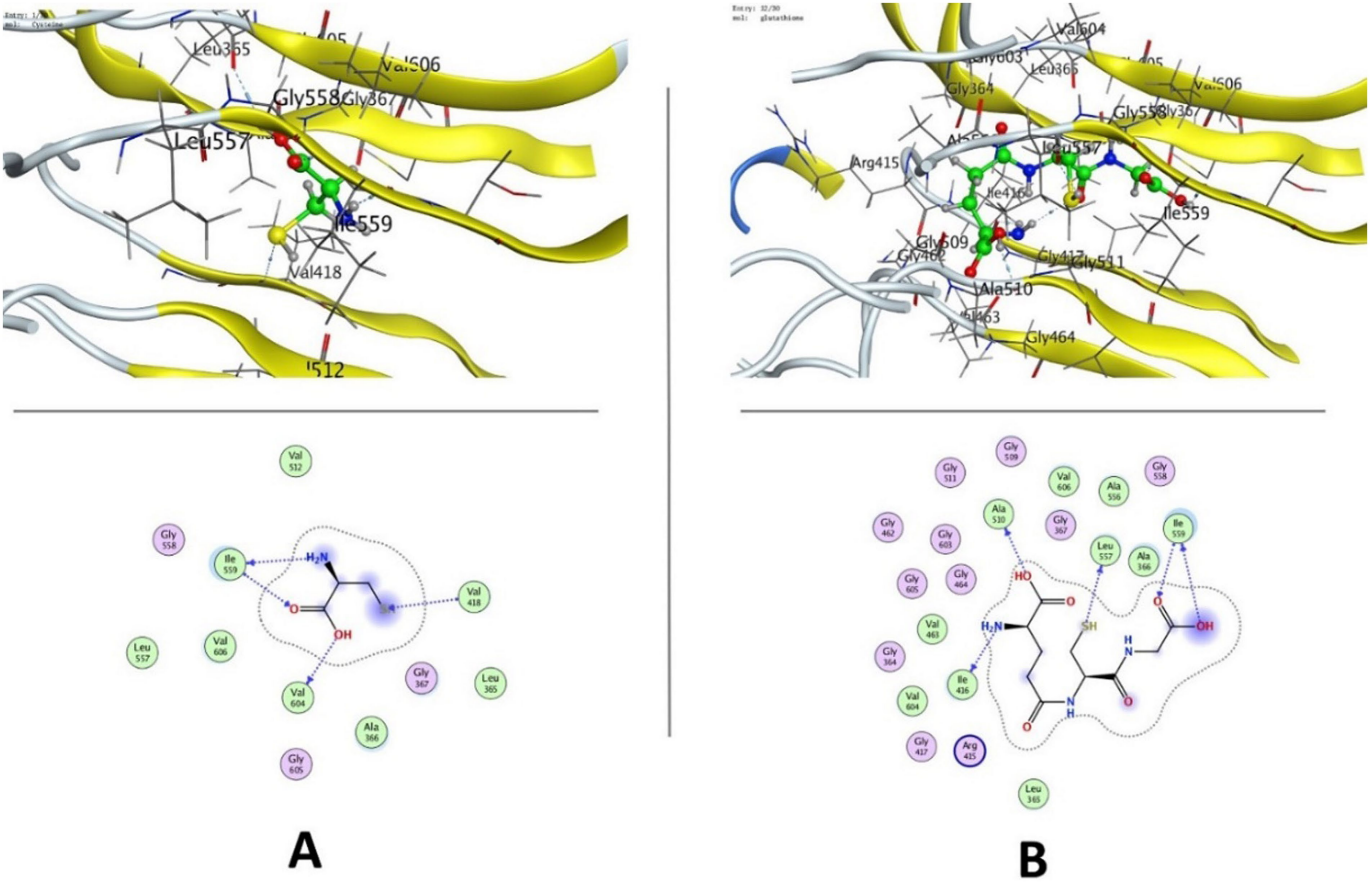
Molecular docking of whey protein components **(A)** cysteine and **(B)** glutathione (GSH) with KEAP1.

To examine binding to KEAP1 (PDB ID: 4IQK), molecular docking of whey-derived bioactives was conducted at the Nrf2-binding site and TGF-*β* Receptor I (ALK5; PDB ID: 1VJY). Cysteine interacted with KEAP1 at −4.21 kcal/mol, forming hydrogen bonds with residues ILE559, VAL604, and VAL418. Glutathione (GSH) exhibited the highest binding affinity at −7.19 kcal/mol, engaging key residues ILE559, LEU557, ILE416, and ALA510, while the positive control sulforaphane bound with −5.25 kcal/mol to GLY367, GLY605, VAL606, and GLY509 (Table 1; Figures 1, 2).

**Table 1:** Molecular docking interactions of whey protein-derived ligands with KEAP1 (PDB ID: 4IQK).

Ligand	Total binding energy (kcal/mol)	Ligand atom	KEAP1 residue (Chain A)	Interaction type	Distance (Å)	E (kcal/mol)
Cysteine	−4.21	N2	ILE559	Hydrogen bond (donor)	3.05	−1.2
		O13	VAL604	Hydrogen bond (donor)	3.20	−2.1
		S10	VAL418	Hydrogen bond (acceptor)	4.41	−1.4
		O12	ILE559	Hydrogen bond (acceptor)	3.20	−1.5
Glutathione (GSH)	−7.19	O9	ILE559	Hydrogen bond (donor)	3.03	−2.1
		S17	LEU557	Hydrogen bond (donor)	3.04	−0.9
		N30	ILE416	Hydrogen bond (donor)	2.89	−1.4
		O34	ALA510	Hydrogen bond (donor)	2.83	−1.2
		O11	ILE559	Hydrogen bond (acceptor)	3.32	−1.2
Sulforaphane (control)	−5.25	S6	GLY367	Hydrogen bond (acceptor)	3.35	−3.5
		S6	GLY605	Hydrogen bond (acceptor)	4.10	−0.8
		S6	VAL606	Hydrogen bond (acceptor)	3.33	−3.1
		O17	GLY509	Hydrogen bond (acceptor)	3.44	−0.7

**Figure 3:**
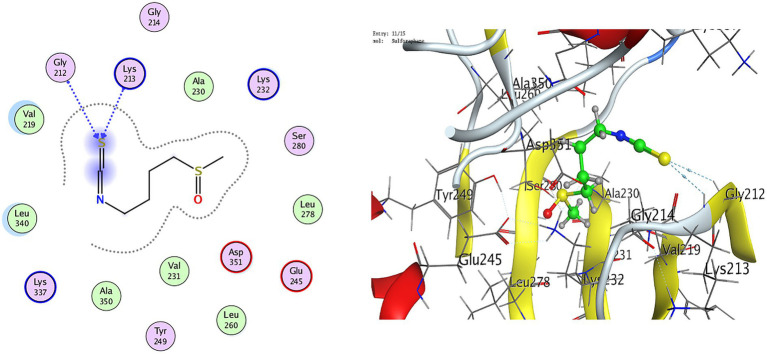
2D, 3D docking of sulforaphane with TGF-*β* receptor I (ALK5; PDB ID: 1VJY).

**Figure 4:**
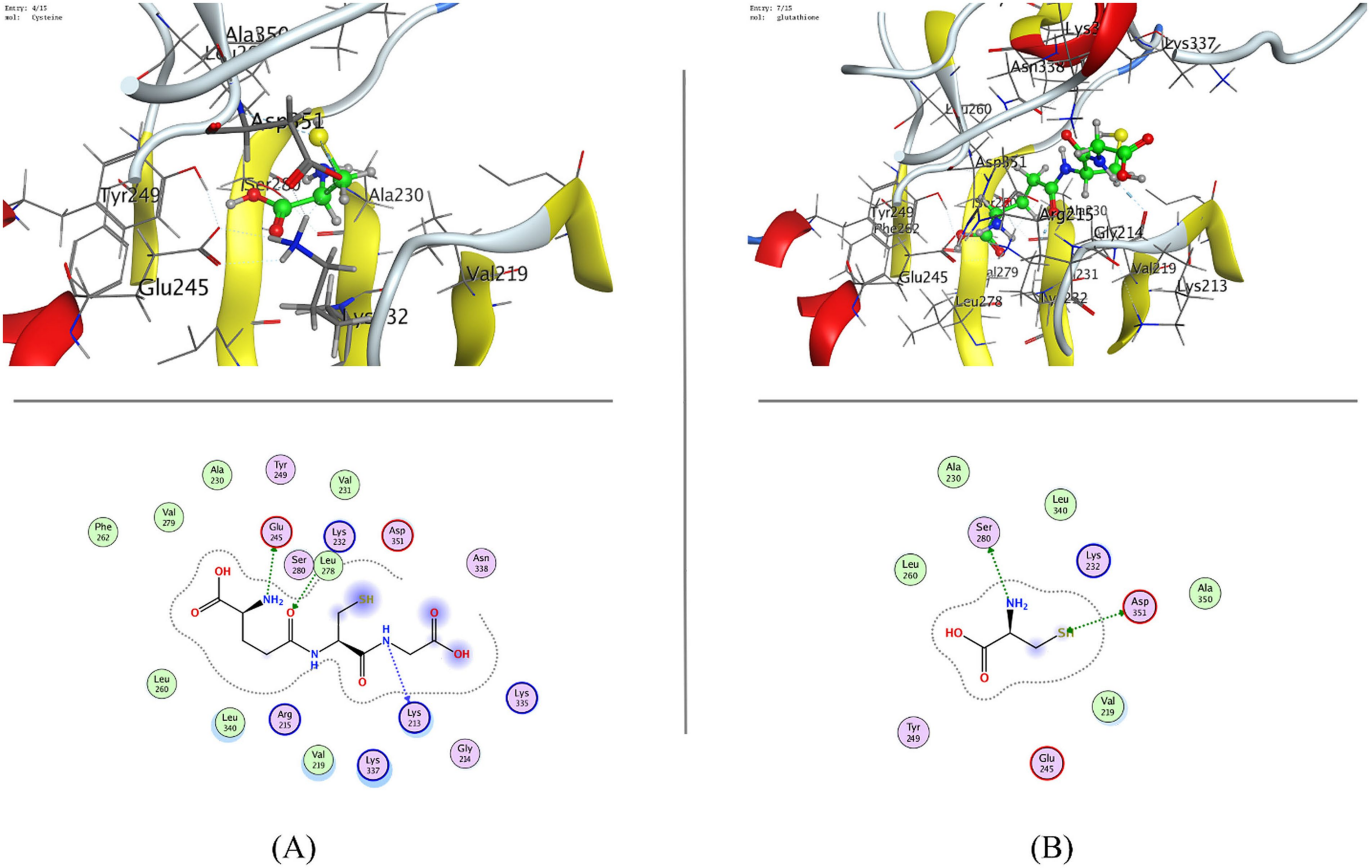
Molecular docking of whey protein components [**(A)** cysteine and **(B)** glutathione (GSH)] with TGF-*β* receptor I (ALK5; PDB ID: 1VJY).

Docking against ALK5 further revealed favorable interactions, where cysteine showed moderate binding (−4.24 kcal/mol) via hydrogen bonding with SER280 and ASP351, whereas GSH exhibited superior affinity (−7.09 kcal/mol) through stable interactions with LYS213, GLU245, and LYS232, surpassing sulforaphane (−5.43 kcal/mol) (Table 2; Figures 3, 4). Collectively, these results indicate stable and favorable binding of whey protein–derived ligands to both KEAP1 and ALK5, with glutathione consistently outperforming the reference inhibitor, highlighting its dual potential in modulating KEAP1-Nrf2 antioxidant signaling and TGF-*β*–mediated inflammatory/fibrotic pathways.

**Table 2:** Molecular docking results of whey protein–derived bioactive components with TGF-*β* receptor I (ALK5; PDB ID: 1VJY).

Ligand	Total binding energy (kcal/mol)	Ligand atom	ALK5 residue (Chain A)	Interaction type	Distance (Å)	E (kcal/mol)
Cysteine	−4.24	N2	SER280	Hydrogen bond (donor)	3.06	−0.8
		S10	ASP351	Hydrogen bond (donor)	3.79	−2.4
		S10	ASP351	Hydrogen bond (acceptor)	3.57	−0.9
Glutathione (GSH)	−7.09	N3	LYS213	Hydrogen bond (donor)	3.21	−2.0
		N30	GLU245	Hydrogen bond (donor)	2.90	−1.4
		O37	LYS232	Hydrogen bond (acceptor)	3.25	−1.1
Sulforaphane (control)	−5.43	S6	GLY212	Hydrogen bond (acceptor)	4.09	−0.8
		S6	LYS213	Hydrogen bond (acceptor)	3.57	−0.8

### Oxidative stress and antioxidant indicators

**Figure 5:**
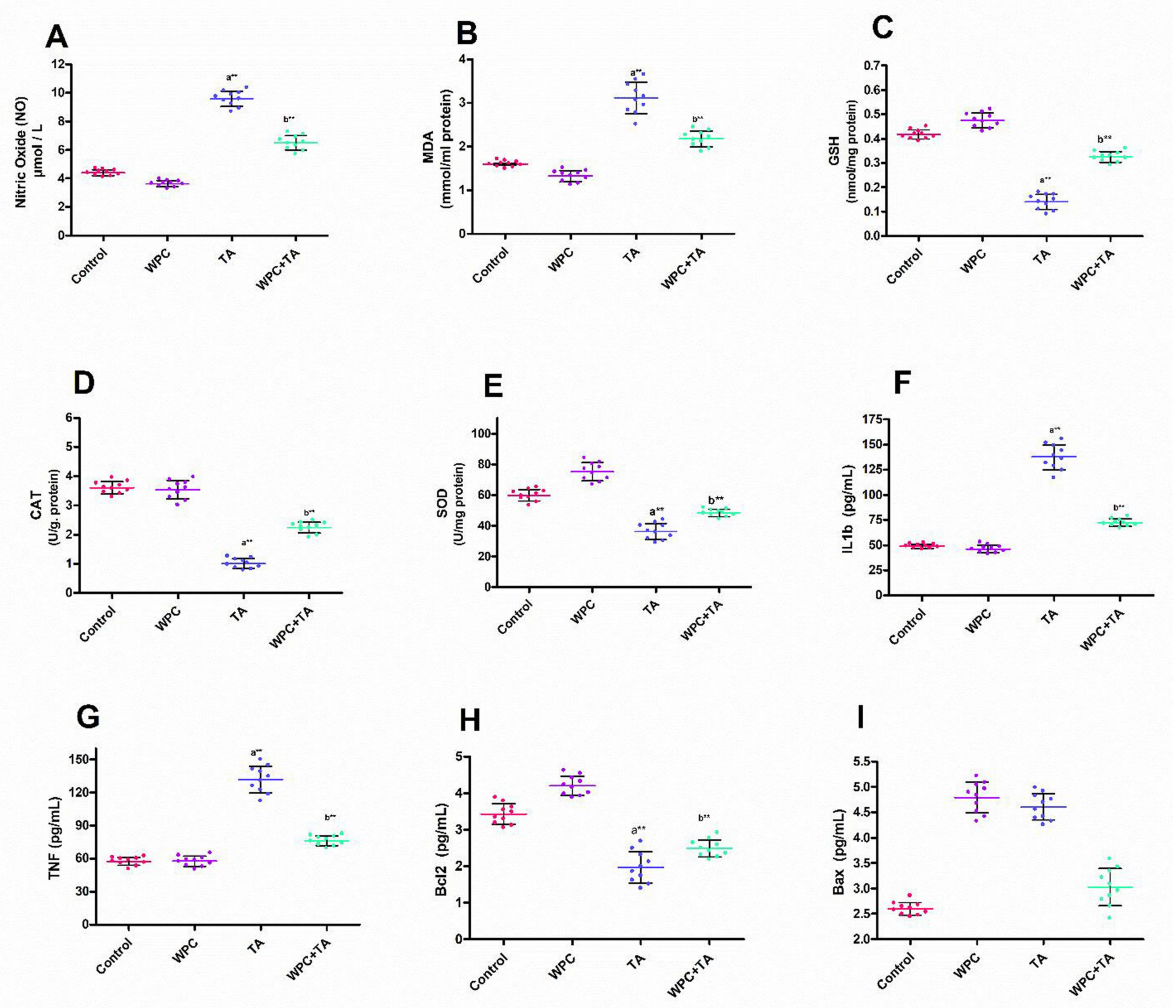
Effects of whey protein (WPC) on intestinal biochemical markers in thioacetamide (TA)-treated rats. **(A)** Nitric oxide (NO) levels. **(B)** Malondialdehyde (MDA) levels. **(C)** Reduced glutathione (GSH) levels. **(D)** Catalase (CAT) activity. **(E)** Superoxide dismutase (SOD) activity. **(F)** Interleukin-1β (IL-1β) levels. **(G)** Tumor necrosis factor-α (TNF-α) levels. **(H)** B-cell lymphoma 2 (Bcl-2) levels. **(I)** Bcl-2-associated X protein (Bax) levels. Data are presented as mean ± SD (n=10 per group). Statistical significance: (a) *p* ≤ 0.05 vs. control; (b) *p* ≤ 0.05 vs. TA group; * *p* ≤ 0.01; ** *p* ≤ 0.001.

The intestinal homogenate levels of NO and MDA across all groups are shown in Figures 5A,B. When equated to the untreated rats, exposure to TA caused a remarkable (*p* ≤ 0.001) rise in both NO and MDA levels. When compared to rats given TA alone, Pre-administration of WPC to rats treated with TA remarkably reduced levels of NO (*p* ≤ 0.001) and MDA (*p* ≤ 0.01). However, intestinal GSH levels and the CAT and SOD activities were considerably (*p* ≤ 0.01) lower in TA-injected rats than in controls, indicating oxidative stress in intestinal tissue. However, WP treatment plus TA considerably (*p* ≤ 0.001) raised GSH levels as well as the activity of both CAT and SOD, compared to rats exposed to TA (Figures 5C–E).

### Apoptotic and inflammatory markers

The levels of apoptotic markers (Bcl-2, Bax) and inflammatory markers (IL-1*β* plus TNF-*α*) in the intestinal homogenate of rats given TA showed a remarkable elevation in the levels of IL-1*β* (*p* ≤ 0.01) in addition to TNF-α (*p* ≤ 0.001), versus the control males. WPC treatment plus TA displayed a substantial (*p* ≤ 0.001) drop in both IL-1*β* plus TNF-α levels relative to TA-injected rats (Figures 5F,G). Furthermore, compared to controls, the TA group caused a remarkable (*p* ≤ 0.001) drop in Bcl-2 levels and an upsurge (*p* ≤ 0.001) in Bax levels. Pre-administration of WPC to the TA group caused a considerable (*p* ≤ 0.001) rise in Bcl-2 levels along with (*p* ≤ 0.001) decline in Bax levels as compared to rats exposed to TA (Figures 5H,I).

### Real-time PCR analysis

**Figure 6:**
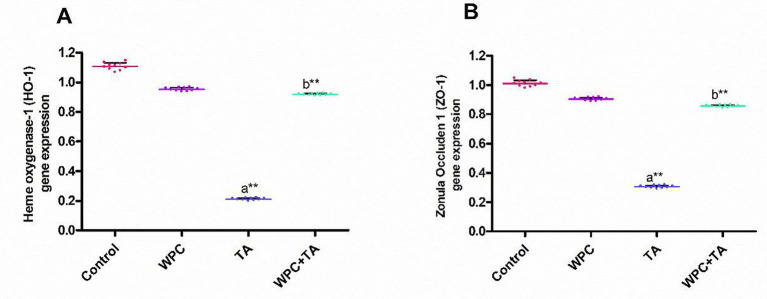
Influence of whey protein supplementation on **(A)** HO-1 and **(B)** ZO-1 gene expression in the intestines of rats exposed to thioacetamide (TA). (a) *p* ≤ 0.05 vs. control; (b) *p* ≤ 0.05 vs. TA group; * *p* ≤ 0.01, ** *p* ≤ 0.001.

Rats treated with TA had considerably (*p* ≤ 0.001) lower intestinal tissue gene expression of HO-1 and ZO-1, comparable to the control group. Relative to the TA-only group, combined WPC and TA treatment resulted in significant upregulation (*p* ≤ 0.001) of intestinal HO-1 and ZO-1. Furthermore, HO-1 and ZO-1 stayed within normal limits, comparable to the control group, when WPC was given in addition to TA, as illustrated in Figures 6A,B.

### Histological investigations

**Figure 7:**
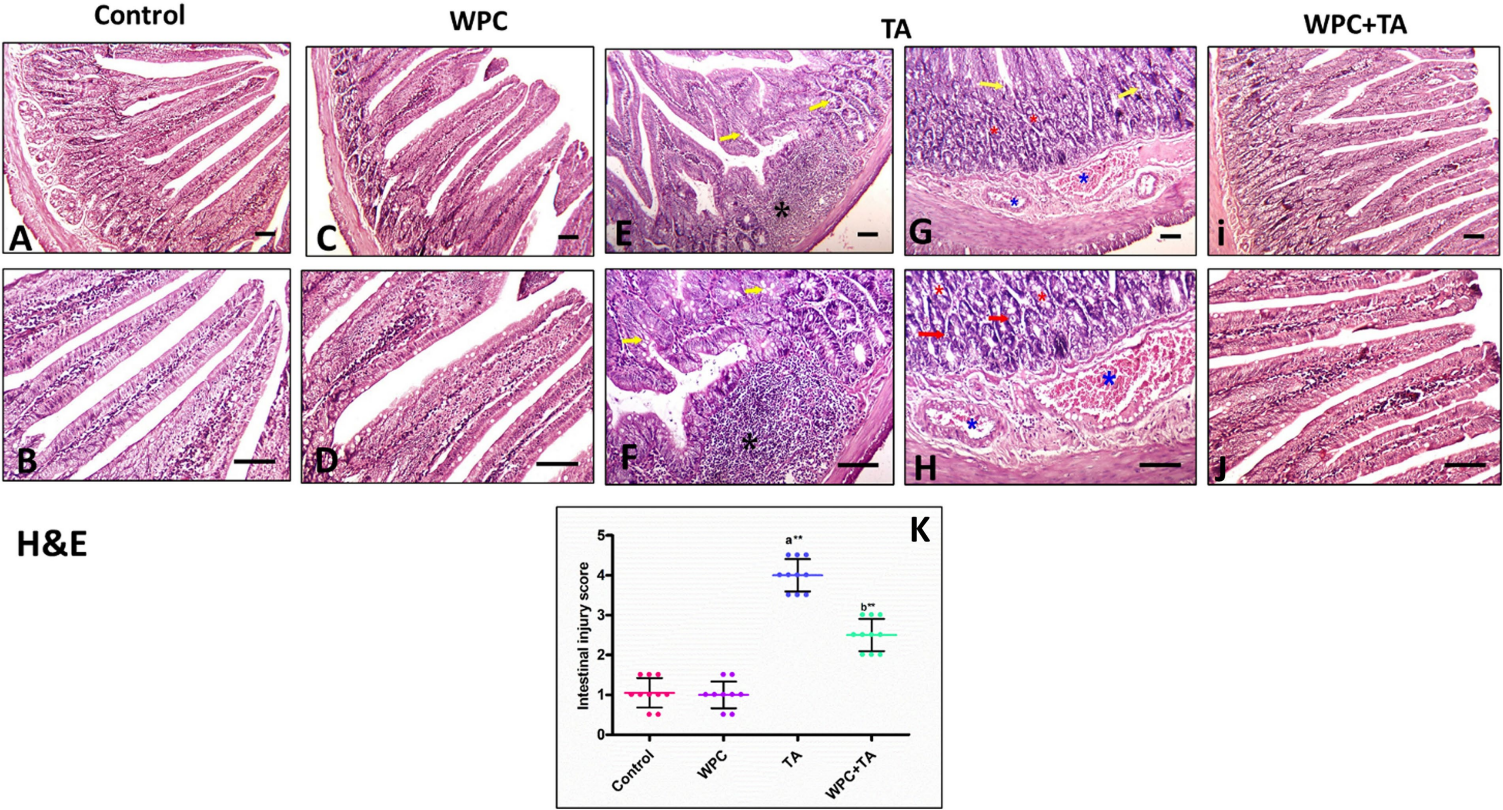
Longitudinal sections of the duodenum illustrating the histopathological alterations among the experimental groups. **(A,B)** Control group showing normal duodenal architecture with intact mucosal epithelium, well-defined villi, normal Brunner’s glands, and absence of inflammatory cell infiltration. **(C,D)** WPC-treated group exhibiting preserved histological structure similar to the control group, with intact villi, normal mucosal epithelium, and normally appearing Brunner’s glands. **(E–H)** TA-treated group demonstrating severe duodenal injury characterized by inflammatory cell infiltration invading submucosa (black asterisk), increased number of goblet cells within Brunner’s glands (yellow arrow), hyperplasia and increased number of Brunner’s glands (red asterisk), single-cell necrosis of the mucosal epithelial lining of crypts (red arrow), and markedly dilated congested blood vessels (blue asterisk). **(I,J)** WPC + TA-treated group showing marked improvement in duodenal histoarchitecture, with approximately normal villous morphology, reduced inflammatory cell infiltration, preservation of mucosal epithelium, and near-normal appearance of Brunner’s glands [H&E staining; bar = 100 μm]. **(K)** Intestinal injury scores (mean ± SEM). (a) *p* ≤ 0.05 vs. control; (b) *p* ≤ 0.05 vs. TA group; * *p* ≤ 0.01, ** *p* ≤ 0.001.

The duodenal histopathology assessed via H&E staining revealed marked structural alterations among the experimental groups (Figure 7). The control group (Figures 7A,B) exhibited intact duodenal architecture with intact mucosal epithelium, well-defined villi, normal Brunner’s glands, and absence of inflammatory cell infiltration, confirming baseline histological integrity. Similarly, the WPC-treated group (Figures 7C,D) exhibited preserved histological structure comparable to the control group, indicating that whey protein concentrate administration alone does not induce any irritative, inflammatory, or degenerative changes in duodenal tissue. In contrast, the TA-treated group (Figures 7E–H) demonstrated severe duodenal injury characterized by extensive inflammatory cell infiltration invading the submucosa (black asterisk), reflecting active inflammation mediated by oxidative stress and pro-inflammatory cytokine release secondary to TA-induced hepatotoxicity. A marked increase in goblet cell numbers within Brunner’s glands (yellow arrow) was observed, indicating a compensatory hypersecretory response to mucosal irritation and an attempt to reinforce the protective mucus barrier. Brunner’s gland hyperplasia (red asterisk) was evident, suggesting regenerative proliferative activity in response to chronic injury. Single-cell necrosis of the mucosal epithelial lining of crypts (red arrow) was detected, signifying early apoptotic cell death and disruption of epithelial integrity, which compromises intestinal barrier function. Additionally, markedly dilated, congested blood vessels (blue asterisk) were present, pointing to vascular dysregulation, increased permeability, and hemodynamic alterations associated with TA toxicity. Notably, the WPC + TA-treated group (Figures 7I,J) showed marked improvement in duodenal histoarchitecture, with approximately normal villous morphology, reduced inflammatory cell infiltration, preservation of mucosal epithelium, and near-normal appearance of Brunner’s glands. Quantitative assessment of the intestinal injury score (Figure 7K) confirmed these histological observations. Duodenal injury score increased significantly in TA-treated rats versus controls (*p* ≤ 0.001), but was markedly reduced by co-treatment with WPC compared to the TA group alone (*p* ≤ 0.01).

### Histochemical investigations

#### Masson’s trichrome

**Figure 8:**
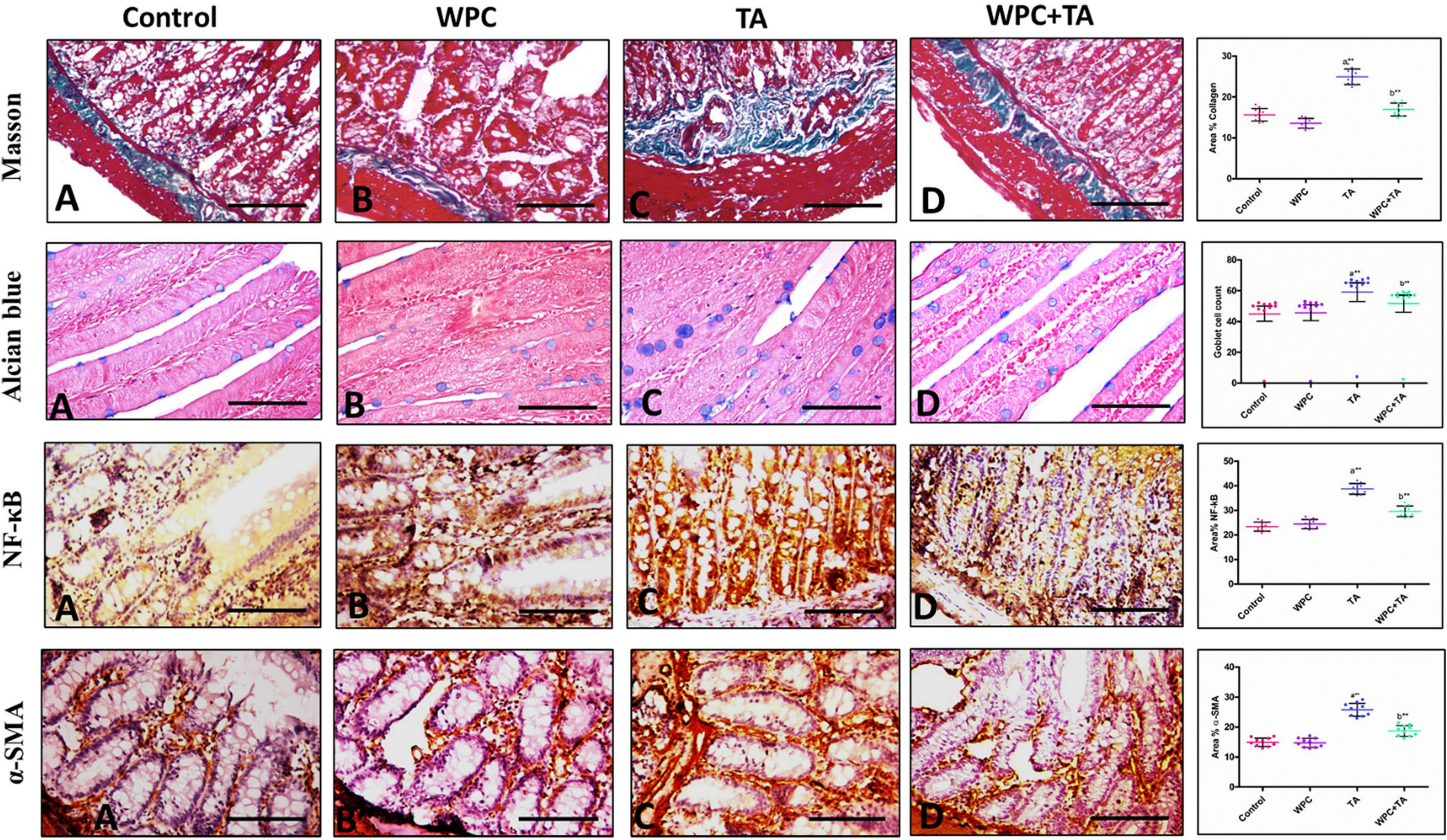
Representative graphs of longitudinal sections of the duodenum illustrating histological, histochemical, and immunohistochemical alterations among the experimental groups. Masson’s trichrome staining (upper panel) demonstrates collagen fiber deposition (blue staining). **(A)** Control group showing normal distribution of collagen fibers within the submucosa. **(B)** WPC-treated group exhibiting collagen content comparable to that of the control group. **(C)** TA-treated group showing marked collagen accumulation and fibrotic changes within the duodenal wall. **(D)** WPC + TA-treated group displaying a notable decline in collagen deposition in contrast to the TA group. The accompanying graph shows quantitative analysis of collagen area percentage, revealing a significant increase in the TA group and a significant reduction following WPC co-treatment. Alcian blue staining (second panel) highlights goblet cells and mucopolysaccharide content. **(A)** Control and **(B)** WPC groups show normal goblet cell distribution along the mucosal lining. **(C)** TA-treated rats exhibit a marked increase in goblet cell count, reflecting mucosal irritation and adaptive response to injury. **(D)** WPC+TA-treated rats show a marked decrease in goblet cell count compared to the TA-injected group, approaching normal levels. Quantitative analysis confirms these observations. NF-κB immunohistochemical staining (third panel) illustrates inflammatory signaling activity. **(A)** Control and **(B)** WPC groups show weak basal NF-κB immunoreactivity. **(C)** TA-treated group demonstrates strong NF-κB expression within the mucosal epithelium and lamina propria, indicating pronounced inflammatory activation. **(D)** WPC+TA-treated group shows markedly reduced NF-κB immunoreactivity. Quantitative analysis confirms a significant increase in the TA group versus controls, which was significantly reduced by WPC co-treatment. α-SMA immunohistochemical staining (lower panel) illustrates myofibroblast activation and fibrogenesis. **(A)** Control group showing minimal α-SMA expression confined to normal smooth muscle layers. **(B)** WPC-treated group exhibiting α-SMA expression comparable to that of the control group. **(C)** TA-treated group showing intense α-SMA immunoreactivity, indicating activation of myofibroblasts and progression of fibrotic remodeling. **(D)** WPC + TA-treated group displaying a notable decrease in α-SMA expression in contrast to the TA group. The accompanying graph shows quantitative analysis of α-SMA-positive area percentage, revealing a significant increase in the TA group and a significant reduction following WPC co-treatment. Significance set at *p* ≤ 0.05. (a) *p* ≤ 0.05 vs. control; (b) *p* ≤ 0.05 vs. TA group; *p ≤ 0.01; **p ≤ 0.001.

In Slides stained with Masson’s trichrome, collagen fibers were found to be distributed normally within the submucosa in the normal controls (Figure 8A). The WPC group (Figure 8B) exhibited collagen content comparable to the control group, indicating that WPC alone does not induce fibrotic changes. In contrast, the TA-treated group (Figure 8C) showed marked collagen accumulation and fibrotic changes within the duodenal wall, confirming significant fibrotic remodeling. Notably, the WPC + TA-treated group (Figure 8D) displayed a notable drop in collagen deposition contrasted to the TA group. These observations were corroborated by collagen percentage analysis, revealing a significant elevation in the TA group relative to controls (*p* ≤ 0.001) and a significant attenuation with WPC co-treatment compared to TA group (*p* ≤ 0.01).

#### Alcian blue

Staining with Alcian blue was performed to assess goblet cells. Control and WPC groups (Figures 8A,B) showed normal goblet cell distribution along the mucosal lining. The TA-treated group (Figure 8C) exhibited a marked increase in goblet cell count, reflecting mucosal irritation and an adaptive secretory response to chemical injury. However, WPC + TA-treated rats (Figure 8D) showed a decrease in goblet cell count compared to the TA-injected rats, approaching normal levels. Quantitative analysis confirmed these observations, indicating that WPC co-treatment effectively normalizes TA-induced goblet cell hyperplasia. Goblet cell quantification revealed distinct, treatment-dependent alterations in the small intestinal mucosa. TA-treated rats showed significantly increased goblet cell counts versus controls (*p* ≤ 0.001), indicating hyperplasia. WPC alone normalized counts to control levels (*p* ≥ 0.05). Co-treatment with WPC partially but significantly attenuated the TA effect, producing intermediate counts lower than TA alone (*p* ≤ 0.001) but still above control and WPC-only groups (*p* ≤ 0.001).

### Immunohistochemical findings

NF-κB immunohistochemical staining was performed to evaluate inflammatory signaling activity. Control and WPC groups (Figures 8A,B) showed weak basal NF-κB immunoreactivity. The TA-treated group (Figure 8C) demonstrated strong NF-κB expression within the mucosal epithelium and lamina propria, indicating pronounced inflammatory activation. In contrast, the WPC + TA-treated group (Figure 8D) showed markedly reduced NF-κB immunoreactivity. Quantification of NF-κB-positive area showed a significant increase in TA-treated rats versus controls (*p* ≤ 0.001), which was markedly reduced by WPC co-treatment (*p* ≤ 0.01 vs. TA), indicating suppression of NF-κB-driven inflammation. *α*-Smooth muscle actin (α-SMA) immunohistochemical staining was performed to assess myofibroblast activation and fibrogenesis. Control and WPC groups (Figures 8A,B) exhibited minimal α-SMA expression confined to normal smooth muscle layers. The TA-treated group (Figure 8C) showed intense α-SMA immunoreactivity, indicating activation of myofibroblasts and progression of fibrotic remodeling. In contrast, WPC + TA-treated rats (Figure 8D) showed decreased α-SMA expression relative to the TA group. Quantification confirmed a significant elevation in the TA group versus controls (*p* ≤ 0.001), which was significantly reduced by WPC co-treatment (*p* ≤ 0.01 vs. TA).

## Discussion

The KEAP1–Nrf2 and TGF-*β*/ALK5 signaling pathways play complementary roles in regulating oxidative stress, inflammation, and tissue remodeling. The KEAP1–Nrf2 axis is a central cellular defense mechanism against oxidative stress, modulating expression of key antioxidants like SOD and CAT (63, 64). Molecular docking at the Nrf2-binding site of KEAP1 (PDB ID: 4IQK) revealed that whey protein–derived bioactive components, particularly glutathione (−7.19 kcal/mol), stably interact with KEAP1, possibly interfering with the KEAP1–Nrf2 interaction in addition to promoting Nrf2 nuclear translocation. Cysteine (−4.21 kcal/mol), although exhibiting moderate binding, serves as a critical precursor for glutathione biosynthesis, thereby reinforcing intracellular antioxidant capacity (65). The recurrent engagement of ILE559, a key residue for Nrf2 recognition, suggests competitive occupation of the Nrf2-binding pocket, providing a molecular basis for the protective effects of WP in TA-induced intestinal oxidative stress (66, 67). In parallel, docking against TGF-*β* Receptor I (ALK5; PDB ID: 1VJY) demonstrated favorable binding of glutathione and cysteine within the kinase domain, with glutathione showing superior affinity compared to the reference inhibitor (68). Given the pivotal role of ALK5 in mediating TGF-*β*–driven inflammatory and fibrotic responses (69, 70), these interactions suggest a potential attenuation of downstream Smad-dependent signaling. In the context of TA-induced intestinal injury, where oxidative stress and TGF-*β* activation synergistically exacerbate inflammation and tissue damage (71, 72), the dual targeting of KEAP1 and ALK5 provides a compelling mechanistic explanation for the observed protective effects of whey protein. Collectively, these results highlight WP as a multifunctional nutraceutical, capable of modulating both antioxidant and pro-inflammatory pathways at the molecular level. By simultaneously activating Nrf2-dependent antioxidant defenses and potentially inhibiting TGF-*β*/ALK5-mediated fibrotic signaling, whey protein can mitigate oxidative stress and inflammation, offering therapeutic potential in TA-induced intestinal toxicity and related oxidative/inflammatory disorders.

It is important to note that TA-induced intestinal damage may arise from both direct and indirect mechanisms. Direct toxicity results from circulating TA metabolites (primarily TA-S-oxide) that reach the intestinal mucosa and induce local oxidative stress. Indirect toxicity occurs secondary to liver injury, where hepatocyte damage triggers the release of pro-inflammatory cytokines (e.g., TNF-*α*, IL-1*β*) and other mediators into the systemic circulation, which then affect distant organs including the intestine (18). Studies have documented that TA-induced liver failure causes bacterial translocation from the gut, increased plasma endotoxin levels, and intestinal oxidative damage (16). While our study was not designed to differentiate between these two pathways, both ultimately converge on common effector mechanisms—oxidative stress, inflammation, apoptosis, and fibrosis—that were effectively mitigated by WPC pretreatment.

The molecular docking data provide a plausible mechanistic explanation for the observed protection by WPC. The high binding affinity of glutathione to KEAP1 (−7.19 kcal/mol) at the Nrf2-binding pocket (involving key residues ILE559, LEU557) suggests that whey-derived components may competitively disrupt KEAP1-Nrf2 interaction, facilitating Nrf2 nuclear translocation (73). This is supported by our finding that WPC treatment upregulated HO-1 gene expression (a canonical Nrf2 target) and restored SOD and CAT activities in TA-exposed rats. Similarly, the favorable docking of glutathione to ALK5 (−7.09 kcal/mol) points to potential inhibition of TGF-*β* signaling, which aligns with our observations of reduced collagen deposition, decreased α-SMA immunoreactivity, and lower inflammatory cytokines. Thus, the in silico results are not merely correlative but directly inform the antioxidant, anti-inflammatory, and anti-fibrotic mechanisms seen *in vivo*.

However, while molecular docking provides valuable insights into potential binding interactions, it is an in silico prediction with inherent limitations. The calculations assume rigid protein structures and do not account for dynamic conformational changes, post-translational modifications, or the complex intracellular environment (e.g., competing proteins, redox state, subcellular compartmentalization) (74). Moreover, binding affinity does not guarantee functional inhibition or activation *in vivo* (75). Therefore, our docking results should be interpreted as hypothesis-generating and complementary to the experimental data, not as proof of a direct molecular mechanism.

In this study, TA exposure dramatically raised NO and MAD levels and decreased GSH levels, as well as CAT and SOD activity in the intestinal tissue homogenate. These results were corroborated by severe histological duodenal alteration. There aren’t enough studies on TA-induced intestinal toxicity, but these findings are consistent with past studies on TA-induced toxicity in other organs caused by increased ROS and an imbalance in the antioxidant system (9, 76). Moreover, TA derivative combination disrupted the antioxidant system and led to the buildup of H2O2; this may be due to the decreased CAT and GSH (77). In addition, Han et al. (78) found that oxidative stress, xenobiotic metabolism, and lipid metabolism were frequently altered genes in the liver and kidney of animals given TA. On the other hand, pre-administration with WPC to TA-treated rats significantly increased GSH levels, CAT and SOD activity, and decreased MDA and NO concentrations. These outcomes were corroborated by improvements in a decrease in the severity grade of intestinal tissue damage. However, after ten days of dosage, WPC had a therapeutic effect on oxidative damage caused by methotrexate in the small intestine by decreasing NO and lipid peroxidation levels and increasing glutathione-S-transferase and superoxide dismutase activity (79). The WP-derived tripeptide Pro-Glu-Trp, a xanthine oxidase inhibitor, reduced hyperuricemia in rats by improving intestinal barrier function (80). Moreover, Sivas et al. (81) found that both WPC intake dosages demonstrated distinct antioxidant effects in the intestinal tissues of rats, with high-dose WP application exhibiting lesser antioxidant capacity in comparison to the ideal WP dose. Interestingly, donkey milk pretreatment and ranitidine significantly reduced ethanol-induced gastric damage by lowering the ulcer score and MDA level and raising GSH in the stomach tissue. These findings demonstrate the gastroprotective and antioxidative properties of donkey milk, as demonstrated by ulcer inhibition (82). Similarly, Humadi et al. (83) found that cow’s milk protected rats from gastrointestinal ulcers by dramatically lowering MDA. Additionally, similar findings have been reported in different tissues by a number of investigations. WP’ antioxidant properties may be explained by their high sulfur-rich amino acid content, especially cysteine, which is a precursor to GSH, the main intracellular antioxidant (84). This is demonstrated in our current study by higher GSH in the WP + TA group, which raises antioxidant enzyme levels and enhances intestinal tissue structure.

Antioxidant enzymes play a vital role in defense mechanisms within the body against oxidative stress. However, Keap1, Nrf2, and HO-1 are crucial antioxidative signaling proteins that help to preserve intestinal cells’ integrity against inflammatory damage and oxidative stress (85). Following oxidative stress and cell injury, HO-1 mRNA is elevated, and Nrf2 may immediately regulate HO-1 inducer activity (86). In this study, to confirm oxidative and antioxidant responses in intestinal tissue, treatment of rats with TA significantly decreased HO-1 gene expression. Our outcomes were in agreement with Đurašević et al. (87), who showed increases in liver oxidative stress caused by TA, as indicated by alterations in SOD activity, GSH levels, and HO-1 expression. However, WPC + TA-treated rats showed a noticeable increase in HO-1 gene expression by enhancing WPC antioxidant components. According to a study on the prevalence of colitis, which found that using WP supplements significantly increased the expression of the HO-1 and Nrf2 genes, while decreasing the inflammatory markers (88). Additionally, soy protein supplements can lower the body’s level of oxidative stress by controlling the expression of the Nrf2 and HO-1 gene (89). The increase in HO-1 gene expression may be due to WPC antioxidant effect of antioxidant-active peptides that may be produced by high-quality protein in the following mechanism: These, once digested *in vivo*, either bind to Keap1 or stop Keap1 and Nrf2 from binding. These peptides then raise the downstream gene HO-1’s transcription and expression, reducing oxidative stress and inflammation (37, 38). This mechanism enables peptides to attach to the Nrf2 site within the Keap1 pocket and provide their antioxidant effects. Consequently, the search for naturally occurring antioxidant peptides requires the discovery of peptides that have the ability to bind to Keap1 (90).

Intestinal inflammation results from the damaged intestinal mucosal barrier, which causes disturbed intestinal flora, abnormal immune regulatory cells, dysfunctional autophagy (91), and increased mucosal permeability (92). Increased mucosal permeability triggers the production of several inflammatory mediators, causing inflammation (93). In this study, rats injected with TA showed elevated IL-1*β* and TNF-*α* levels. However, pro-inflammatory cytokines, intestinal macrophages, and intestinal lumen mucosa epithelial cells all produce more when NF-κB is activated in the intestinal lumen. Intestinal inflammation is more severe when there are more cells with active NF-κB labeling. Accordingly, this inflammatory elevation was confirmed by increased NF-κB immunoreactivity in intestinal tissue and was in line with our previous study (94). Also, Abdel-Rahman et al. (43) found that TA treatment increased NF-κB and IL-1*β* expression in hepatic tissue along with an increase in IL-1*β* and TNF-α levels (95). On the other hand, WPC and TA treatment positively decreased the levels of intestinal inflammatory indicators (IL-1*β* and TNF-α), which were confirmed by decreased NF-κB immunoreactivity in intestinal tissue. However, WPI treatment decreased TNF-α and IL-6 in patients with diabetes (96), and lowered IL-1*β*, IL-6, IL-10, and TNF-α in diabetic mice (97). Also, rectal administration of WPI improved colon histopathological changes in rats with ulcerative colitis in rats, with significant reductions in inflammatory markers (IL-10, IL-6, NF-κB, NF-κB, COX-2 and TNF-α), as well as a decrease in HO-1 protein (88). The same findings were reported in many studies on animal models of colitis; intestinal inflammation was reduced by dietary high-quality protein (98), and donkey WP (40). The decreased inflammatory indicators levels in intestinal tissue may be due to WP inhibition of NF-κB, a key regulator of intestinal inflammation, which contributes to its anti-inflammatory effect (39). This is confirmed in our recent work by increased HO-1 gene expression. WP may protect against NF-κB-induced inflammation by enhancing HO-1 expression and stimulating Nrf2 signaling (32). According to research on animals, giving IBD rats a daily dose of WP can dramatically lower the expression of NF-κB in their colon (40, 41).

Intestinal barrier function is the intestine’s capacity to control the passage of water and nutrients while inhibiting the passage of harmful antigens, bacteria, and toxins. The epithelial cells and mucin are two primary parts of the intestine’s barrier, and they perform a vital role in forming physical and chemical barriers. Any disturbance in intact epithelial cells and the mucus layer disrupts the function of the intestinal barrier, leading to increased permeability that permits the passage of pro-inflammatory substances, causing chronic intestinal inflammation (7). However, tight junctions (TJs) are significant cellular components that maintain the structural integrity of tissues and barrier efficiency. Tight junction proteins (Claudins, Occludin, and ZO-1) establish a continual intercellular barrier to exogenous substances in the gut (99). The integrity of the intestinal mucosal barrier is compromised when intestinal tight junction protein expression or distribution is aberrant, which increases permeability and results in intestinal inflammation, allergies, and other health problems. This hypothesis was consistent with the results of the present intestinal histopathological alterations and the rise in inflammatory markers, which were supported by a decrease in ZO-1 in the intestine of rats given TA. These results were in line with previous TA toxicity studies (18, 19, 44, 100), which reported that TA exposure increased oxidative stress, along with decreasing the levels of gut tight junction and adherens junction proteins. On the other hand, WPC and TA treatments effectively improved intestinal barrier function as evidenced by increasing the intestinal ZO-1 expression. This was supported by a dramatic improvement in duodenal histoarchitecture, which included practically normal villous form, reduced inflammatory cell infiltration, and the preservation of mucosal epithelium. The results were also accompanied by considerable decreases in inflammatory markers (IL-1*β* and TNF-*α*). Similar studies found that WPC improved the expression of intestinal TJ proteins, including ZO-1, in the lipopolysaccharide-treated piglets (101), and in both Caco-2 and HT29-MTX intestinal cell lines (102). Additionally, in animals with colitis, WPI dramatically reduced TNF-α expression in the colon and increased intestinal mucosal permeation via regulating tight junction protein dispersion (88). *In vitro* study, WPC enhanced barrier function in (Caco-2) human intestinal cells (25).

Goblet cells in the intestinal villi and crypts create mucin, which protects and lubricates the intestinal mucosa. The quantity of goblet cells indirectly represents the ability to secrete mucus (103). The intestinal mucins are glycoproteins released by goblet cells of the intestinal epithelium. They serve as a barrier between the intestinal epithelium and bacteria and regulate the transit and level of ions, water, and other immune-mediated molecules (104). The primary component of intestinal mucus is mucin 2 (MUC2), which connects disulfide bonds to form the intestinal mucus layer’s skeleton (105). In the present study, the duodenal mucosa of rats given TA has more goblet cells; similar results were reported by Al-Rawi et al. (15). Unlike our results, Zhang et al. (106) found that cirrhotic rats had fewer and smaller ileal goblet cells despite enhanced epithelial proliferation, a sign of compromised epithelial homeostasis. It has been reported that, in response to inflammation, injury, or infection, the intestine may activate a repair mechanism that raises the goblet cells as a protective response for restoring the intestinal barrier, while prolonged stress can result in a decline in goblet cells and impaired mucosal barrier function (107). Administration of TA and WP effectively ameliorated goblet cell counts in the duodenal tissue. These results demonstrate that WPC maintains mucosal homeostasis under baseline conditions and provides a moderating effect against TA-driven goblet cell proliferation; however, most research focuses on the importance of WP in mucin production. In this regard, food-derived peptides have opioid receptor agonist qualities that may directly activate epithelial goblet cells to control the production of mucin (108). Also, a previous study found that milk protein hydrolysates can cause intestinal goblet cells to secrete a significant amount of mucin by activating the opioid receptors (109). Moreover, in the colitis-animal model, WP and soy protein can increase colonic MUC2 synthesis to decrease mucosal damage (110).

Intestinal fibrosis is a pathological healing process marked by the ongoing production of pro-fibrogenic cytokines, proteolytic enzymes, and growth factors in response to continuing inflammation. Chronic intestinal inflammation activates fibroblasts and myofibroblasts, which results in an increase in collagen and other extracellular matrix components (111). These mediators trigger extracellular matrix remodeling, which results in tissue deterioration, ultimately causing intestinal stenosis, loss of function, and irreparable organ damage (42). Intestinal fibrosis is caused by the complex interactions between mesenchymal cells, fibroblasts, myofibroblasts, smooth muscle cells, macrophages, and TGF-*β* signaling molecule (112). Intracellular proteins known as *α*-SMA and collagen type 1 maturation enzymes are used to identify fibroblasts and myofibroblasts in intestinal fibrosis (113). In this study, histochemical examination demonstrated that TA-treated rats had greater collagen fiber content; this result was corroborated by an increase in immunoreactivity of *α*-SMA and NF-κ*β* immunoreactivity. According to similar findings, TA exposure increased the amounts of liver fibrosis-indicative proteins, including collagen-1, TGF-*β*, and α-SMA (44). A plausible explanation for intestinal fibrosis could arise from either chronic inflammation and pro-fibrotic cytokines IL-1, 4, 6, 13, and 17, or the major regulator driving fibrosis in numerous organs, TGF-*β* signaling molecule (114). Also, TA-treated mice showed a substantial collagen deposition in liver tissue and elevated TGF-*β* expression (115). Moreover, TA-treated rats showed elevated expressions of NF-κB and collagen I (95), activated NF-κB and TGF-*β*, which in turn activated fibrogenic proteins in heart tissue (43). On the contrary, treatment of TA and WPC successfully lowered α-SMA immunoreactivity and collagen deposition in intestinal tissue. Collagen synthesis, transformation, and proliferation in fibrosis may have been inhibited by the decreased levels of TGF-*β*1 and NF-κB seen in this study. Similar outcomes were noted when lactoferrin, a byproduct of WP, was administered (116). Our earlier study (94) demonstrated that WPC treatment significantly regulated cardiac fibrosis induced by TA via decreasing NF-κ*β* immunoreactivity and TGF-*β* expression. Similarly, the favorable docking of glutathione to ALK5 (−7.09 kcal/mol) points to potential inhibition of TGF-*β* signaling, which aligns with our observations of reduced collagen deposition, decreased α-SMA immunoreactivity, and lower inflammatory cytokines.

Oxidative stress-induced apoptosis is a crucial contributor to cell death. However, in humans or rats exposed to alcohol, elevated ROS levels cause enterocytes to have decreased amounts of adhesion and tight-junction factors, which promote apoptosis (117). In the current biochemical data of apoptotic markers, TA-treated rats showed an increase in intestinal Bax levels while decreasing Bcl-2 levels. These results may be due to the overproduction of ROS, which disrupted the mitochondrial membrane, where Bax activation promotes mitochondrial membrane permeability by forming pores or channels, ultimately releasing cytochrome c into the cytoplasm. When cytochrome C is released, caspases are activated, leading to cell disintegration (118). In TA-induced hepatic fibrosis, TA decreased Bcl-2 while increasing the amounts of hydrolyzed caspase-3 and p53 in rats (119), and decreased Bcl-2 and increased Bax gene expression in mice (95), and in zebrafish larvae (120). In addition, TA treatment either increased the expression of the caspase-8 gene, while reducing the expression of the Bax gene (121). Similarly, Rungratanawanich et al. (44) found that TA exposure increased histological alterations, which were associated with the increased protein levels of the apoptosis marker, cleaved (active)-caspase 3. On the other hand, administration of TA and WPC effectively suppressed apoptosis, as evidenced by a rise in the level of Bcl-2 and a reduction in the Bax. Moreover, in the H₂O₂-instigated PC12 cell line under oxidative stress, WPI enhanced antioxidant enzyme activities, reduced apoptosis by downregulating Bax and upregulating Bcl-2, and prevented caspase-3 activation (122). Likewise, camel WP significantly reduced the increased caspases 3 and 9 in heat-exposed mice (123), and the Bax/Bcl-2 in diabetic mice (124). In the neuroprotective impact of WP, Chang et al. (125) found that WPI regulated the Bax/Bcl2 in HT22 cells brought on by H2O2-induced oxidative stress. Additionally, WP supplements reduced cytochrome C and caspases 8 and 9 in rotenone-treated rats to induce Parkinson’s disease (126). The present outcomes showed that WPC’s ability to regulate the mitochondrial apoptotic process, which may be responsible for its protective impact against oxidative stress.

Whey proteins are generally regarded as a safe supplement because few adverse effects have been documented in earlier research (84). Although regular dietary use of active peptides is generally considered safe, overdose may have adverse effects, leading to toxicology (84). Therefore, it’s crucial to appropriately modify the dosage and duration of the WP supplement where, the optimal dosage of whey protein improves antioxidant effects in the liver, kidney, and intestinal tissue more than the high dose. Also, both WPC intake dosages demonstrated distinct antioxidant effects in the intestinal tissues of rats, with high-dose WP application exhibiting lesser antioxidant capacity in comparison to the ideal WP dose (81). However, some worries about a WP overdose include potential negative consequences. However, Yiğit Ziolkowski et al. (127) found that adding 200 mg/kg of WPI to a control diet for 8 weeks resulted in greater liver damage scores. Another study found that healthy rats given WPC-80 at a dosage of 0.3 g/kg for 21 days experienced hepatocellular damage; animals given a greater dose (0.5 g/kg) of WPC-80 showed more severe cellular impairment (128). Further studies on how high-quality proteins impact gastrointestinal system health is required to understand these changes and the underlying causes. Consequently, additional information is required to look into the usage of various doses in both short-term and long-term research about the safety concerns.

## Limitations and future studies

The limitations of this study should be acknowledged. First, molecular docking is an in silico prediction that assumes rigid protein structures and does not account for dynamic conformational changes, post-translational modifications, or the complex intracellular environment; therefore, binding affinity does not guarantee functional activity *in vivo*. Second, direct protein-level validation of key signaling pathways (e.g., Western blotting for Nrf2, Keap1, and phosphorylated Smad proteins) is lacking. Although our ELISA and immunohistochemical data provide indirect evidence of protein expression, they do not confirm the activation status of these pathways at the post-translational level.

Future studies should address these limitations by: (1) experimentally validating the predicted docking interactions using surface plasmon resonance, cellular thermal shift assays, or genetic knockdown of Nrf2/Keap1; and (2) incorporating Western blot analysis to measure the expression levels of Nrf2, Keap1, and phosphorylated Smad2/3, thereby confirming the involvement of these pathways at the translational level.

## Conclusion

In conclusion, it is well recognized that intestinal toxicity can result from an excess of reactive oxygen species caused by TA exposure. However, TA induced intestinal injury, duodenal fibrosis, goblet cell hyperplasia, inflammatory activation, and myofibroblast differentiation. Additionally, WPC’s antioxidant qualities may help reduce oxidative stress and boost the antioxidant defenses, hence avoiding oxidative intestinal damage. The results of this investigation demonstrated unequivocally that WPC’s protective properties improved the rats’ intestinal damage caused by TA at the molecular, histological, and biochemical levels by reducing systemic inflammation, apoptosis, and fibrosis; strengthening the gut barrier function by upregulating Z0-1 gene expression; and modulating oxidative stress by upregulating H0-1 gene expression. Additionally, the molecular docking data suggested possible interactions by confirming the antioxidant peptides’ binding affinity to TGF-*β*, Nrf2, and Keap1. Therefore, WPC may be a viable treatment option for intestinal damage. To fully comprehend the signaling pathways and the underlying mechanisms of WPC’s antioxidant action and how dietary proteins regulate the goblet cell number and its mucin production, a more specific mechanism is necessary before WPC can be used as a protective dietary supplement for intestinal health. Overall, these findings emphasize the importance of WPC in regulating the TA-induced duodenal injury through its antioxidant, anti-inflammatory, and cytoprotective properties.

## Data Availability

The original contributions presented in the study are included in the article/Supplementary material, further inquiries can be directed to the corresponding author.

## References

[1] Camilleri á Madsen K Spiller R Van Meerveld B Verne G. Intestinal barrier function in health and gastrointestinal disease. Neurogastroenterol Motil. (2012) 24:503–12. doi: 10.1111/j.1365-2982.2012.01921.x, 22583600 PMC5595063

[2] Bischoff SC Barbara G Buurman W Ockhuizen T Schulzke J-D Serino M . Intestinal permeability-a new target for disease prevention and therapy. BMC Gastroenterol. (2014) 14:189. doi: 10.1186/s12876-014-0189-7, 25407511 PMC4253991

[3] Suzuki T. Regulation of intestinal epithelial permeability by tight junctions. Cell Mol Life Sci. (2013) 70:631–59. doi: 10.1007/s00018-012-1070-x, 22782113 PMC11113843

[4] Li DK Chaudhari SN Lee Y Sojoodi M Adhikari AA Zukerberg L . Inhibition of microbial deconjugation of micellar bile acids protects against intestinal permeability and liver injury. Sci Adv. (2022) 8:eabo2794. doi: 10.1126/sciadv.abo2794, 36026454 PMC9417178

[5] Xu S Li X Zhang S Qi C Zhang Z Ma R . Oxidative stress gene expression, DNA methylation, and gut microbiota interaction trigger Crohn's disease: a multi-omics Mendelian randomization study. BMC Med. (2023) 21:179. doi: 10.1186/s12916-023-02878-8, 37170220 PMC10173549

[6] Wang Y Chen Y Zhang X Lu Y Chen H. New insights in intestinal oxidative stress damage and the health intervention effects of nutrients: a review. J Funct Foods. (2020) 75:104248. doi: 10.1016/j.jff.2020.104248

[7] Liang S-j Wang X-q. Deoxynivalenol induces intestinal injury: insights from oxidative stress and intestinal stem cells. Environ Sci Pollut Res. (2023) 30:48676–85. doi: 10.1007/s11356-023-26084-4, 36856999

[8] Cao S Wu H Wang C Zhang Q Jiao L Lin F . Diquat-induced oxidative stress increases intestinal permeability, impairs mitochondrial function, and triggers mitophagy in piglets. J Anim Sci. (2018) 96:1795–805. doi: 10.1093/jas/sky104, 29562342 PMC6140957

[9] Türkmen NB Hande Y Taşlidere A Şahin Y Çiftçi O. The ameliorate effects of nerolidol on thioacetamide-induced oxidative damage in heart and kidney tissue. Turk J Pharm Sci. (2022) 19:1. doi: 10.4274/tjps.galenos.(2021).30806 35227035 PMC8892551

[10] Yang H Zhang H Tian L Guo P Liu S Chen H . Curcumin attenuates lupus nephritis by inhibiting neutrophil migration via PI3K/AKT/NF-κB signalling pathway. Lupus Sci Med. (2024) 11:e001220. doi: 10.1136/lupus-2024-001220, 39053932 PMC11284931

[11] Ghanim AM Younis NS Metwaly HA. Vanillin augments liver regeneration effectively in Thioacetamide induced liver fibrosis rat model. Life Sci. (2021) 286:120036. doi: 10.1016/j.lfs.2021.120036, 34637793

[12] Su W Tai Y Tang S-H Ye Y-T Zhao C Gao J-H . Celecoxib attenuates hepatocyte apoptosis by inhibiting endoplasmic reticulum stress in thioacetamide-induced cirrhotic rats. World J Gastroenterol. (2020) 26:4094–107. doi: 10.3748/wjg.v26.i28.4094, 32821072 PMC7403803

[13] Yang HY Kim KS Lee YH Park JH Kim J-H Lee S-Y . Dendropanax morbifera ameliorates thioacetamide-induced hepatic fibrosis via TGF-β1/Smads pathways. Int J Biol Sci. (2019) 15:800–11. doi: 10.7150/ijbs.30356, 30906211 PMC6429015

[14] Ortega MA Torres MI Fernandez MI Rios A Sanchez-Pozo A Gil A. Hepatotoxic agent thioacetamide induces biochemical and histological alterations in rat small intestine. Dig Dis Sci. (1997) 42:1715–23. doi: 10.1023/A:1018817600238, 9286239

[15] Al-Rawi MM. The protective role of melatonin on small intestine after thioacetamide intoxication in rats. Arab Gulf J Sci Res. (1989) 2007:25.

[16] Harputluoğlu MMM Temel İ Demirel U Seçkin Y Aladağ M Otlu B . Methylprednisolone prevents bacterial translocation in thioacetamide-induced liver failure in rats. Turk J Gastroenterol. (2017) 28:394–400. doi: 10.5152/tjg.2017.1775, 28776498

[17] Al-Qaisi TS Jabbar AA Ubeid MH Al-Qaaneh AM Mothana RA Hawwal MF . A traditional medicinal food Arum rupicola (Kardeh) ameliorates thioacetamide-induced hepatotoxicity in animal model. Ital J Food Sci. (2024) 36:208. doi: 10.15586/ijfs.v36i4.2723

[18] Rungratanawanich W Qu Y Holmes A Kaplowitz N Song B-J. Early oxidative protein modifications and gut damage/leakiness contribute to drug-induced acute liver failure. Clin Mol Hepatol. (2025) 32:e29. doi: 10.3350/cmh.2025.0748, 40755007 PMC12835785

[19] Caliskan AR Gul M Yılmaz I Otlu B Uremis N Uremis MM . Effects of larazotide acetate, a tight junction regulator, on the liver and intestinal damage in acute liver failure in rats. Hum Exp Toxicol. (2021) 40:S693–701. doi: 10.1177/09603271211058882 34791921

[20] Pan Q Liu X Ye F Niu Y. Gastrointestinal function and pathologic changes in rats of acute liver failure caused by thioacetamide. J Xi'an Jiaotong Univ. (2008) 29:66–9.

[21] Giromini C Cheli F Rebucci R Baldi A. Invited review: dairy proteins and bioactive peptides: modeling digestion and the intestinal barrier. J Dairy Sci. (2019) 102:929–42. doi: 10.3168/jds.2018-15163, 30591343

[22] Cava E Padua E Campaci D Bernardi M Muthanna FM Caprio M . Investigating the health implications of whey protein consumption: a narrative review of risks, adverse effects, and associated health issues. Healthcare. (2024) 12:246. doi: 10.3390/healthcare12020246, 38255133 PMC10815430

[23] Price D Jackson KG Lovegrove JA Givens DI. The effects of whey proteins, their peptides and amino acids on vascular function. Nutr Bull. (2022) 47:9–26. doi: 10.1111/nbu.12543, 36045079

[24] Teixeira FJ Santos HO Howell SL Pimentel GD. Whey protein in cancer therapy: a narrative review. Pharmacol Res. (2019) 144:245–56. doi: 10.1016/j.phrs.2019.04.019, 31005617

[25] Willems E Purba A Savoian MS Hefer C Maes E Ulluwishewa D. Effects of whey protein treatment in an in vitro intestinal cell model following oxidative stress or inflammatory challenge. Int Dairy J. (2025) 164:106187. doi: 10.1016/j.idairyj.2025.106187

[26] Mann B Athira S Sharma R Kumar R Sarkar P. Bioactive peptides from whey proteins. In: Deeth HC, Bansal N, editors. Whey Proteins: From Milk to Medicine. London, United Kingdom: Academic Press. (2019). p. 519–47.

[27] Cheng W Li C Xiao F He J Liu L Niu H . Elucidating binding mechanisms of caffeic acid and resveratrol by beta-lactoglobulin: insights into hydrophobic interactions and complex formation. Food Hydrocoll. (2024) 146:109269. doi: 10.1016/j.foodhyd.2023.109269

[28] Heine WE Klein PD Reeds PJ. The importance of α-lactalbumin in infant nutrition. J Nutr. (1991) 121:277–83. doi: 10.1093/jn/121.3.277, 2002399

[29] Giblin L Yalçın AS Biçim G Krämer AC Chen Z Callanan MJ . Whey proteins: targets of oxidation, or mediators of redox protection. Free Radic Res. (2019) 53:1136–52. doi: 10.1080/10715762.2019.1632445, 31510814

[30] Keleku-Lukwete N Suzuki M Yamamoto M. An overview of the advantages of KEAP1-NRF2 system activation during inflammatory disease treatment. Antioxid Redox Signal. (2018) 29:1746–55. doi: 10.1089/ars.2017.7358, 28899203

[31] Kansanen E Jyrkkänen H-K Levonen A-L. Activation of stress signaling pathways by electrophilic oxidized and nitrated lipids. Free Radic Biol Med. (2012) 52:973–82. doi: 10.1016/j.freeradbiomed.2011.11.038, 22198184

[32] Chen F Xiao M Hu S Wang M. Keap1-Nrf2 pathway: a key mechanism in the occurrence and development of cancer. Front Oncol. (2024) 14:1381467. doi: 10.3389/fonc.2024.1381467, 38634043 PMC11021590

[33] Li Q Wang J. The effect of protein nutritional support on inflammatory bowel disease and its potential mechanisms. Nutrients. (2024) 16:2302. doi: 10.3390/nu16142302, 39064745 PMC11280054

[34] Rosaneli C Bighetti A Antonio M Carvalho J Sgarbieri V. Efficacy of a whey protein concentrate on the inhibition of stomach ulcerative lesions caused by ethanol ingestion. J Med Food. (2002) 5:221–8. doi: 10.1089/109662002763003375, 12639397

[35] Zhao J Huang Y Yu X. Effects of nutritional supplement and resistance training for sarcopenia in patients with inflammatory bowel disease: a randomized controlled trial. Medicine. (2022) 101:e30386. doi: 10.1097/MD.0000000000030386, 36042627 PMC9410600

[36] Ferreiro B Llopis-Salinero S Lardies B Granados-Colomina C Milà-Villarroel R. Clinical and nutritional impact of a semi-elemental hydrolyzed whey protein diet in patients with active Crohn's disease: a prospective observational study. Nutrients. (2021) 13:3623. doi: 10.3390/nu13103623, 34684624 PMC8538212

[37] Rebollo-Hernanz M Kusumah J Bringe NA Shen Y de Mejia EG. Peptide release, radical scavenging capacity, and antioxidant responses in intestinal cells are determined by soybean variety and gastrointestinal digestion under simulated conditions. Food Chem. (2023) 405:134929. doi: 10.1016/j.foodchem.2022.134929

[38] Ma Y Xu J Guo R Teng G Chen Y Xu X. In vitro gastrointestinal model for the elderly: effect of high hydrostatic pressure on protein structures and antioxidant activities of whey protein isolate. Food Biosci. (2023) 52:102452. doi: 10.1016/j.fbio.2023.102452

[39] Mussbacher M Salzmann M Brostjan C Hoesel B Schoergenhofer C Datler H . Cell type-specific roles of NF-κB linking inflammation and thrombosis. Front Immunol. (2019) 10:85. doi: 10.3389/fimmu.2019.00085, 30778349 PMC6369217

[40] Li M Li Q Abdlla R Chen J Yue X Quek SY. Donkey whey proteins ameliorate dextran sulfate sodium-induced ulcerative colitis in mice by downregulating the S100A8-TRAF6-NF-κB axis-mediated inflammatory response. Food Sci Human Wellness. (2023) 12:1809–19. doi: 10.1016/j.fshw.2023.02.045

[41] Li Y Zhang Y Tuo Y You H Li J Wang L . Quinoa protein and its hydrolysate ameliorated DSS-induced colitis in mice by modulating intestinal microbiota and inhibiting inflammatory response. Int J Biol Macromol. (2023) 253:127588. doi: 10.1016/j.ijbiomac.2023.127588, 37875182

[42] Li Y Xu F Fang Y Cui Y Zhu Z Wu Y . Inflammation-fibrosis interplay in inflammatory bowel disease: mechanisms, progression, and therapeutic strategies. Front Pharmacol. (2025) 16:1530797. doi: 10.3389/fphar.2025.1530797, 40093318 PMC11906429

[43] Abdel-Rahman RF Fayed HM Asaad GF Ogaly HA Hessin AF Salama AA . The involvement of TGF-β1/FAK/α-SMA pathway in the antifibrotic impact of rice bran oil on thioacetamide-induced liver fibrosis in rats. PLoS One. (2021) 16:e0260130. doi: 10.1371/journal.pone.0260130, 34965258 PMC8716044

[44] Rungratanawanich W LeFort KR Cho YE Li X Song BJ. Melatonin prevents thioacetamide-induced gut leakiness and liver fibrosis through the gut-liver axis via modulating Sirt1-related deacetylation of gut junctional complex and hepatic proteins. J Pineal Res. (2024) 76:e13007. doi: 10.1111/jpi.13007, 39269018 PMC11480967

[45] Garg G Singh S Singh AK Rizvi SI. Whey protein concentrate supplementation protects rat brain against aging-induced oxidative stress and neurodegeneration. Appl Physiol Nutr Metab. (2018) 43:437–44. doi: 10.1139/apnm-2017-0578, 29199432

[46] Reagan-Shaw S Nihal M Ahmad N. Dose translation from animal to human studies revisited. FASEB J. (2008) 22:659–61. doi: 10.1096/fj.07-9574LSF, 17942826

[47] Bank, RPD. Crystal Structure of cpd 16 Bound to Keap1 Kelch Domain (2013). Available online at: https://www.rcsb.org/structure/4IQK (Accessed March 3, 2026).

[48] Corrochano AR Buckin V Kelly PM Giblin L. Invited review: whey proteins as antioxidants and promoters of cellular antioxidant pathways. J Dairy Sci. (2018) 101:4747–61. doi: 10.3168/jds.2017-13618, 29605324

[49] Forman HJ Zhang H Rinna A. Glutathione: overview of its protective roles, measurement, and biosynthesis. Mol Asp Med. (2009) 30:1–12. doi: 10.1016/j.mam.2008.08.006, 18796312 PMC2696075

[50] Houghton CA. Sulforaphane: its "coming of age" as a clinically relevant nutraceutical in the prevention and treatment of chronic disease. Oxidative Med Cell Longev. (2019) 2019:2716870. doi: 10.1155/2019/2716870, 31737167 PMC6815645

[51] Trott O Olson A. Software news and update AutoDock Vina: improving the speed and accuracy of docking with a new scoring function. *Effic Optim multithreading*. J Comput Chem. (2009) 31:455–61. doi: 10.1002/jcc.21334, 19499576 PMC3041641

[52] Bradford MM. A rapid and sensitive method for the quantitation of microgram quantities of protein utilizing the principle of protein-dye binding. Anal Biochem. (1976) 72:248–54. doi: 10.1016/0003-2697(76)90527-3, 942051

[53] Ramos-Vara JA Kiupel M Baszler T Bliven L Brodersen B Chelack B . Suggested guidelines for immunohistochemical techniques in veterinary diagnostic laboratories. J Vet Diagn Invest. (2008) 20:393–413. doi: 10.1177/104063870802000401, 18599844

[54] Archer S. Measurement of nitric oxide in biological models. FASEB J. (1993) 7:349–60. doi: 10.1096/fasebj.7.2.8440411, 8440411

[55] Beutler E Duron O Kelly M. Colorimetric method for determination of glutathione reduced. J Lab Clin Med. (1963) 61:882. 13967893

[56] Nishikimi M Rao NA Yagi K. The occurrence of superoxide anion in the reaction of reduced phenazine methosulfate and molecular oxygen. Biochem Biophys Res Commun. (1972) 46:849–54. doi: 10.1016/S0006-291X(72)80218-3, 4400444

[57] Aebi H. Catalase in vitro. Methods Enzymol. (1984) 105:121–6. doi: 10.1016/S0076-6879(84)05016-3, 6727660

[58] Livak KJ Schmittgen TD. Analysis of relative gene expression data using real-time quantitative PCR and the 2− ΔΔCT method. Methods. (2001) 25:402–8. doi: 10.1006/meth.2001.1262 11846609

[59] Bancroft JD Gamble M. Theory and Practice of Histological Techniques. London: Churchill Livingstone (2008).

[60] Sgarbi FC Bertini F de M Tera T Cavalcante ASR. Morphology of collagen fibers and elastic system fibers in actinic cheilitis. Indian J Dent Res. (2010) 21:518–22. doi: 10.4103/0970-9290.74224, 21187617

[61] Benight NM Stoll B Olutoye OO Holst JJ Burrin DG. GLP-2 delays but does not prevent the onset of necrotizing enterocolitis in preterm pigs. J Pediatr Gastroenterol Nutr. (2013) 56:623–30. doi: 10.1097/MPG.0b013e318286891e, 23343934 PMC3976429

[62] Itoh H Beck PL Inoue N Xavier R Podolsky DK. A paradoxical reduction in susceptibility to colonic injury upon targeted transgenic ablation of goblet cells. J Clin Invest. (1999) 104:1539–47. doi: 10.1172/JCI6211, 10587517 PMC409855

[63] Serafini MM Catanzaro M Fagiani F Simoni E Caporaso R Dacrema M . Modulation of Keap1/Nrf2/ARE signaling pathway by curcuma-and garlic-derived hybrids. Front Pharmacol. (2020) 10:1597. doi: 10.3389/fphar.2019.01597, 32047434 PMC6997134

[64] Yu C Xiao JH. The Keap1-Nrf2 system: a mediator between oxidative stress and aging. Oxidative Med Cell Longev. (2021) 2021:6635460. doi: 10.1155/2021/6635460, 34012501 PMC8106771

[65] Pant A Dasgupta D Tripathi A Pyaram KJI. Beyond antioxidation: keap1-Nrf2 in the development and effector functions of adaptive immune cells. Immunohorizons. (2023) 7:288–98. doi: 10.4049/immunohorizons.2200061, 37099275 PMC10579846

[66] Huenchuguala S Segura-Aguilar JJA. Natural compounds that activate the KEAP1/Nrf2 signaling pathway as potential new drugs in the treatment of idiopathic Parkinson's disease. Antioxidants. (2024) 13:1125. doi: 10.3390/antiox13091125, 39334784 PMC11428591

[67] Ngo V Duennwald MLJA. Nrf2 and oxidative stress: a general overview of mechanisms and implications in human disease. Antioxidants. (2022) 11:2345. doi: 10.3390/antiox11122345, 36552553 PMC9774434

[68] Gao Y Yu Q Zhu H Zhang Y Yang HJCS. The trilateral crosstalk of TGF β1-ROS-Nrf2 axis in fibrosis: decoding mechanistic networks for precision therapeutics. Cell Signal. (2025):112327. doi: 10.1016/j.cellsig.2025.112327 41407174

[69] Gong Y Yang YJL. Activation of Nrf2/AREs-mediated antioxidant signalling, and suppression of profibrotic TGF-β1/Smad3 pathway: a promising therapeutic strategy for hepatic fibrosis-a review. Life Sci. (2020) 256:117909. doi: 10.1016/j.lfs.2020.117909, 32512009

[70] Churchman AT Anwar AA Li FY Sato H Ishii T Mann GE . Transforming growth factor-β1 elicits Nrf2-mediated antioxidant responses in aortic smooth muscle cells. J Cell Mol Med. (2009) 13:2282–92. doi: 10.1111/j.1582-4934.2009.00874.x, 19674192 PMC6529974

[71] Wen Z Liu W Li X Chen W Liu Z Wen J . A protective role of the NRF2-Keap1 pathway in maintaining intestinal barrier function. Oxidative Med Cell Longev. (2019) 2019:1759149. doi: 10.1155/2019/1759149, 31346356 PMC6617875

[72] Khan MZ Li S Ullah A Li Y Abohashrh M Alzahrani FM . Therapeutic agents targeting the Nrf2 signaling pathway to combat oxidative stress and intestinal inflammation in veterinary and translational medicine. Vet Sci. (2025) 13:25. doi: 10.3390/vetsci13010025, 41600681 PMC12846341

[73] Itoh K Wakabayashi N Katoh Y Ishii T Igarashi K Engel JD . Keap1 represses nuclear activation of antioxidant responsive elements by Nrf2 through binding to the amino-terminal Neh2 domain. Genes Dev. (1999) 13:76–86. doi: 10.1101/gad.13.1.76, 9887101 PMC316370

[74] Ferreira LG Dos Santos RN Oliva G Andricopulo AD. Molecular docking and structure-based drug design strategies. Molecules. (2015) 20:13384–421. doi: 10.3390/molecules200713384, 26205061 PMC6332083

[75] Chen Y-C. Beware of docking! Trends Pharmacol Sci. (2015) 36:78–95. doi: 10.1016/j.tips.2014.12.001, 25543280

[76] Omar S Abd ElAziz MM Mady MM. Protective role of quercetin in preventing thioacetamide induced heart and lung injury in adult male albino rat. Egypt J Histol. (2022) 45:1156–1172. doi: 10.21608/ejh.2022.145573.1711

[77] Jorgačević B Stanković S Filipović J Samardžić J Vučević D Radosavljević T. Betaine modulating MIF-mediated oxidative stress, inflammation and fibrogenesis in thioacetamide-induced nephrotoxicity. Curr Med Chem. (2022) 29:5254–67. doi: 10.2174/0929867329666220408102856, 35400322

[78] Han H-Y Park S-M Ko J-W Oh J-H Kim SK Kim T-W. Integrated transcriptomic analysis of liver and kidney after 28 days of thioacetamide treatment in rats. Toxicol Res. (2023) 39:201–11. doi: 10.1007/s43188-022-00156-y, 37008694 PMC10050285

[79] Yılmaz S Tufan E Sivas GG Gökmen BG Dursun E Özbeyli D . The effect of whey proteins on the brain and small intestine nitric oxide levels: protein profiles in methotrexate-induced oxidative stress. Experimed. (2022) 12:113–8. doi: 10.26650/experimed.1189898

[80] Qi X Ma Y Guan K Zhao L Ma Y Wang R. Whey protein peptide pro-glu-trp ameliorates hyperuricemia by enhancing intestinal uric acid excretion, modulating the gut microbiota, and protecting the intestinal barrier in rats. J Agric Food Chem. (2024) 72:2573–84. doi: 10.1021/acs.jafc.3c00984, 38240209

[81] Sivas G Tufan E Yilmaz Karaoglu S Gurel Gokmen B Dursun E Ozbeyli D . Effects of high-dose whey protein concentrate intake on hepatorenal and intestinal tissues. Pharm J. (2024) 1:1–10. doi: 10.62482/pmj.3

[82] Sami M Azizi S Kheirandish R Ebrahimnejad H Alizadeh S. Protective effects of donkey Milk on ethanol-induced gastric ulcer in rat. Vet Med Sci. (2025) 11:e70156. doi: 10.1002/vms3.70156, 39665798 PMC11636306

[83] Humadi AT Mahdi AN Hussein AA. Effect of cow's milk and glutathione as a protective factor on physiological and histological parameters in male rats with gastric ulcers. J Pioneer Med Sci. (2025) 14:45–52. doi: 10.47310/jpms2025141021

[84] Yiğit A Bielska P Cais-Sokolińska D Samur G. Whey proteins as a functional food: health effects, functional properties, and applications in food. J Am Nutr Assoc. (2023) 42:758–68. doi: 10.1080/27697061.2023.2169208, 36725371

[85] Gu R Cui T Guo Y Luan Y Wang X Liu R . Angiotensin-(1-7) ameliorates intestinal barrier dysfunction by activating the Keap1/Nrf2/HO-1 signaling pathway in acute pancreatitis. Mol Biol Rep. (2023) 50:5991–6003. doi: 10.1007/s11033-023-08544-9, 37269386

[86] Zhu H Jia Z Misra BR Zhang L Cao Z Yamamoto M . Nuclear factor E2-related factor 2-dependent myocardiac cytoprotection against oxidative and electrophilic stress. Cardiovasc Toxicol. (2008) 8:71–85. doi: 10.1007/s12012-008-9016-0, 18463988

[87] Đurašević S Pejić S Grigorov I Nikolić G Mitić-Ćulafić D Dragićević M . Effects of C60 fullerene on thioacetamide-induced rat liver toxicity and gut microbiome changes. Antioxidants. (2021) 10:911. doi: 10.3390/antiox10060911, 34199786 PMC8226855

[88] Tunc N Sahin A Demirel U Artas G Sahin K Bahcecioglu İH . Favourable effects of whey protein on acetic acid-induced ulcerative colitis in a rat model. Arch Med Sci. (2021) 18:1617. doi: 10.5114/aoms/105839 36457969 PMC9710278

[89] Zhao X Gao J Hogenkamp A Knippels LM Garssen J Bai J . Selenium-enriched soy protein has antioxidant potential via modulation of the NRF2-HO1 signaling pathway. Foods. (2021) 10:2542. doi: 10.3390/foods10112542, 34828827 PMC8623322

[90] Guo H Zang C Zheng L Ding L Yang W Ren S . Novel antioxidant peptides from fermented whey protein by *Lactobacillus rhamnosus* B2-1: separation and identification by in vitro and *in silico* approaches. J Agric Food Chem. (2024) 72:23306-19. doi: 10.1021/acs.jafc.4c07531 39392363 PMC11505895

[91] Sergiu MI Elena MM Marilena M Andrada D Emanuela PR. Microbiota: the missing link in the etiology of inflammatory bowel disease. J Mind Med Sci. (2020) 7:5. doi: 10.22543/7674.71.P2933

[92] Tamburini B La Manna MP La Barbera L Mohammadnezhad L Badami GD Shekarkar Azgomi M . Immunity and nutrition: the right balance in inflammatory bowel disease. Cells. (2022) 11:455. doi: 10.3390/cells11030455, 35159265 PMC8834599

[93] Goldszmid RS Trinchieri G. The price of immunity. Nat Immunol. (2012) 13:932–8. doi: 10.1038/ni.2422, 22990891

[94] Almohawes ZN Okail HA Al-Megrin WA El-Khadragy MF Ibrahim MA Fathalla AS . The cardioprotective effect of whey protein against thioacetamide-induced toxicity through its antioxidant, anti-inflammatory, and anti-apoptotic effects in male albino rats. Front Vet Sci. (2025) 12:1590722. doi: 10.3389/fvets.2025.1590722, 40458758 PMC12127417

[95] Chen X Ding C Liu W Liu X Zhao Y Zheng Y . Abscisic acid ameliorates oxidative stress, inflammation, and apoptosis in thioacetamide-induced hepatic fibrosis by regulating the NF-кB signaling pathway in mice. Eur J Pharmacol. (2021) 891:173652. doi: 10.1016/j.ejphar.2020.173652, 33069671

[96] Derosa G D'angelo A Maffioli P. Change of some oxidative stress parameters after supplementation with whey protein isolate in patients with type 2 diabetes. Nutrition. (2020) 73:110700. doi: 10.1016/j.nut.2019.110700, 32065880

[97] Badr G Badr BM Mahmoud MH Mohany M Rabah DM Garraud O. Treatment of diabetic mice with undenatured whey protein accelerates the wound healing process by enhancing the expression of MIP-1α, MIP-2, KC, CX3CL1 and TGF-β in wounded tissue. BMC Immunol. (2012) 13:32. doi: 10.1186/1471-2172-13-32, 22708778 PMC3676145

[98] Ahn E Jeong H Kim E. Differential effects of various dietary proteins on dextran sulfate sodium-induced colitis in mice. Nutr Res Pract. (2022) 16:700–15. doi: 10.4162/nrp.(2022).16.6.700, 36467764 PMC9702549

[99] Hu C Xiao K Luan Z Song J. Early weaning increases intestinal permeability, alters expression of cytokine and tight junction proteins, and activates mitogen-activated protein kinases in pigs. J Anim Sci. (2013) 91:1094–101. doi: 10.2527/jas.2012-5796, 23230104

[100] Yang L Bian X Wu W Lv L Li Y Ye J . Protective effect of *Lactobacillus salivarius* Li01 on thioacetamide-induced acute liver injury and hyperammonaemia. Microb Biotechnol. (2020) 13:1860–76. doi: 10.1111/1751-7915.13629, 32652882 PMC7533332

[101] Xiao K Jiao L Cao S Song Z Hu C Han X. Whey protein concentrate enhances intestinal integrity and influences transforming growth factor-β1 and mitogen-activated protein kinase signalling pathways in piglets after lipopolysaccharide challenge. Br J Nutr. (2016) 115:984–93. doi: 10.1017/S0007114515005085, 26810899

[102] Arbizu S Chew B Mertens-Talcott SU Noratto G. Commercial whey products promote intestinal barrier function with glycomacropeptide enhanced activity in downregulating bacterial endotoxin lipopolysaccharides (LPS)-induced inflammation in vitro. Food Funct. (2020) 11:5842–52. doi: 10.1039/D0FO00487A, 32633745

[103] Odenwald MA Turner JR. The intestinal epithelial barrier: a therapeutic target? Nat Rev Gastroenterol Hepatol. (2017) 14:9–21. doi: 10.1038/nrgastro.2016.169, 27848962 PMC5554468

[104] Paone P Cani PD. Mucus barrier, mucins and gut microbiota: the expected slimy partners? Gut. (2020) 69:2232–43. doi: 10.1136/gutjnl-2020-322260, 32917747 PMC7677487

[105] Zhang B Fan X Du H Zhao M Zhang Z Zhu R . Foodborne carbon dot exposure induces insulin resistance through gut microbiota dysbiosis and damaged intestinal mucus layer. ACS Nano. (2023) 17:6081–94. doi: 10.1021/acsnano.3c01005, 36897192

[106] Zhang L Tai Y Tang S Zhao C Tong H Gao J . Compromised ileal mucus barrier due to impaired epithelial homeostasis caused by notch1 signaling in cirrhotic rats. Dig Dis Sci. (2021) 66:131–42. doi: 10.1007/s10620-020-06178-6, 32144600

[107] Wu M-H Chen L-W Chen J-H Lai C-W. Goblet cell-mediated pathway: a major contributor to increased intestinal permeability in Streptozotocin-induced type 1 diabetic mice. Int J Mol Sci. (2025) 26:8890. doi: 10.3390/ijms26188890, 41009456 PMC12469469

[108] Martinez-Maqueda D Miralles B De Pascual-Teresa S Reverón I Muñoz R Recio I. Food-derived peptides stimulate mucin secretion and gene expression in intestinal cells. J Agric Food Chem. (2012) 60:8600–5. doi: 10.1021/jf301279k, 22916966

[109] Martínez-Maqueda D Miralles B Ramos M Recio I. Effect of β-lactoglobulin hydrolysate and β-lactorphin on intestinal mucin secretion and gene expression in human goblet cells. Food Res Int. (2013) 54:1287–91. doi: 10.1016/j.foodres.2012.12.029

[110] Jayatilake S Arai K Kumada N Ishida Y Tanaka I Iwatsuki S . The effect of oral intake of low-temperature-processed whey protein concentrates on colitis and gene expression profiles in mice. Foods. (2014) 3:351–68. doi: 10.3390/foods3020351, 28234324 PMC5302365

[111] Rieder F Mukherjee PK Massey WJ Wang Y Fiocchi C. Fibrosis in IBD: from pathogenesis to therapeutic targets. Gut. (2024) 73:854–66. doi: 10.1136/gutjnl-2023-329963, 38233198 PMC10997492

[112] Koliaraki V Prados A Armaka M Kollias G. The mesenchymal context in inflammation, immunity and cancer. Nat Immunol. (2020) 21:974–82. doi: 10.1038/s41590-020-0741-2, 32747813

[113] Lovisa S Genovese G Danese S. Role of epithelial-to-mesenchymal transition in inflammatory bowel disease. J Crohns Colitis. (2019) 13:659–68. doi: 10.1093/ecco-jcc/jjy201, 30520951

[114] Yun S-M Kim S-H Kim E-H. The molecular mechanism of transforming growth factor-β signaling for intestinal fibrosis: a mini-review. Front Pharmacol. (2019) 10:162. doi: 10.3389/fphar.2019.00162, 30873033 PMC6400889

[115] Dwivedi DK Jena G. Glibenclamide protects against thioacetamide-induced hepatic damage in Wistar rat: investigation on NLRP3, MMP-2, and stellate cell activation. Naunyn Schmiedeberg's Arch Pharmacol. (2018) 391:1257–74. doi: 10.1007/s00210-018-1540-2, 30066023

[116] Chen R Huang L Zheng W Zhang M Xin Z Liu L . Lactoferrin ameliorates myocardial fibrosis by inhibiting inflammatory response via the AMPK/NF-κB pathway in aged mice. J Funct Foods. (2022) 93:105106. doi: 10.1016/j.jff.2022.105106

[117] Cho Y-E Yu L-R Abdelmegeed MA Yoo S-H Song B-J. Apoptosis of enterocytes and nitration of junctional complex proteins promote alcohol-induced gut leakiness and liver injury. J Hepatol. (2018) 69:142–53. doi: 10.1016/j.jhep.2018.02.005, 29458168 PMC6008177

[118] Morse PT Arroum T Wan J Pham L Vaishnav A Bell J . Phosphorylations and Acetylations of cytochrome c control mitochondrial respiration, mitochondrial membrane potential, energy, ROS, and apoptosis. Cells. (2024) 13:493. doi: 10.3390/cells13060493, 38534337 PMC10969761

[119] Lee YH Son JY Kim KS Park YJ Kim HR Park JH . Estrogen deficiency potentiates thioacetamide-induced hepatic fibrosis in Sprague-Dawley rats. Int J Mol Sci. (2019) 20:3709. doi: 10.3390/ijms20153709, 31362375 PMC6696236

[120] Wang K Deng Y Zhang J Cheng B Huang Y Meng Y . Toxicity of thioacetamide and protective effects of quercetin in zebrafish (*Danio rerio*) larvae. Environ Toxicol. (2021) 36:2062–72. doi: 10.1002/tox.23323, 34227734

[121] Eraky SM El-Kashef DH El-Sherbiny M El-Magd NFA. Naringenin mitigates thioacetamide-induced hepatic encephalopathy in rats: targeting the JNK/Bax/caspase-8 apoptotic pathway. Food Funct. (2023) 14:1248–58. doi: 10.1039/D2FO03470K, 36625308

[122] Jin M-M Zhang L Yu H-X Meng J Sun Z Lu R-R. Protective effect of whey protein hydrolysates on H2O2-induced PC12 cells oxidative stress via a mitochondria-mediated pathway. Food Chem. (2013) 141:847–52. doi: 10.1016/j.foodchem.2013.03.076, 23790857

[123] Ramadan NK Badr G Abdel-Tawab HS Ahmed SF Mahmoud MH. Camel whey protein enhances lymphocyte survival by modulating the expression of survivin, bim/bax, and cytochrome C and restores heat stress-mediated pathological alteration in lymphoid organs. Iran J Basic Med Sci. (2018) 21:896–904. doi: 10.22038/IJBMS.2018.27584.6729, 30524689 PMC6272073

[124] Sayed LH Badr G Omar HM Abd El-Rahim AM Mahmoud MH. Camel whey protein improves oxidative stress and histopathological alterations in lymphoid organs through Bcl-XL/Bax expression in a streptozotocin-induced type 1 diabetic mouse model. Biomed Pharmacother. (2017) 88:542–52. doi: 10.1016/j.biopha.2017.01.076, 28129627

[125] Chang YB Jung E-J Jo K Suh HJ Choi H-S. Neuroprotective effect of whey protein hydrolysate containing leucine-aspartate-isoleucine-glutamine-lysine on HT22 cells in hydrogen peroxide-induced oxidative stress. J Dairy Sci. (2024) 107:2620–32. doi: 10.3168/jds.2023-24284, 38101744

[126] Zarei P Amirpour-Najafabadi B Sam-Sani P Sakhaie MH Sadegh M. Protective effect of whey protein supplement against rotenone induced motor dysfunction in a rat model of Parkinson disease. Adv Biomed Res. (2024) 13:93. doi: 10.4103/abr.abr_178_23 39717252 PMC11665149

[127] Yiğit Ziolkowski A Şenol N Aslankoç R Samur G. Whey protein supplementation reduced the liver damage scores of rats fed with a high fat-high fructose diet. PLoS One. (2024) 19:e0301012. doi: 10.1371/journal.pone.0301012, 38573884 PMC10994406

[128] Żebrowska-Gamdzyk M Maciejczyk M Zalewska A Guzińska-Ustymowicz K Tokajuk A Car H. Whey protein concentrate WPC-80 intensifies glycoconjugate catabolism and induces oxidative stress in the liver of rats. Nutrients. (2018) 10:1178. doi: 10.3390/nu10091178, 30154356 PMC6164859

