# Supplementary material for: In silico molecular docking and in vivo evidence of whey protein concentrate-mediated protection against thioacetamide-induced intestinal toxicity in male albino rats via modulation of oxidative stress, inflammation, apoptosis, and fibrosis
Source: Front Nutr. 2026 May 13;13:1817773. doi: 10.3389/fnut.2026.1817773 (PMC13212499; doi:10.3389/fnut.2026.1817773)
Supplement: Supplementary file 2 [file Presentation_1.PPTX]

## Slide 1
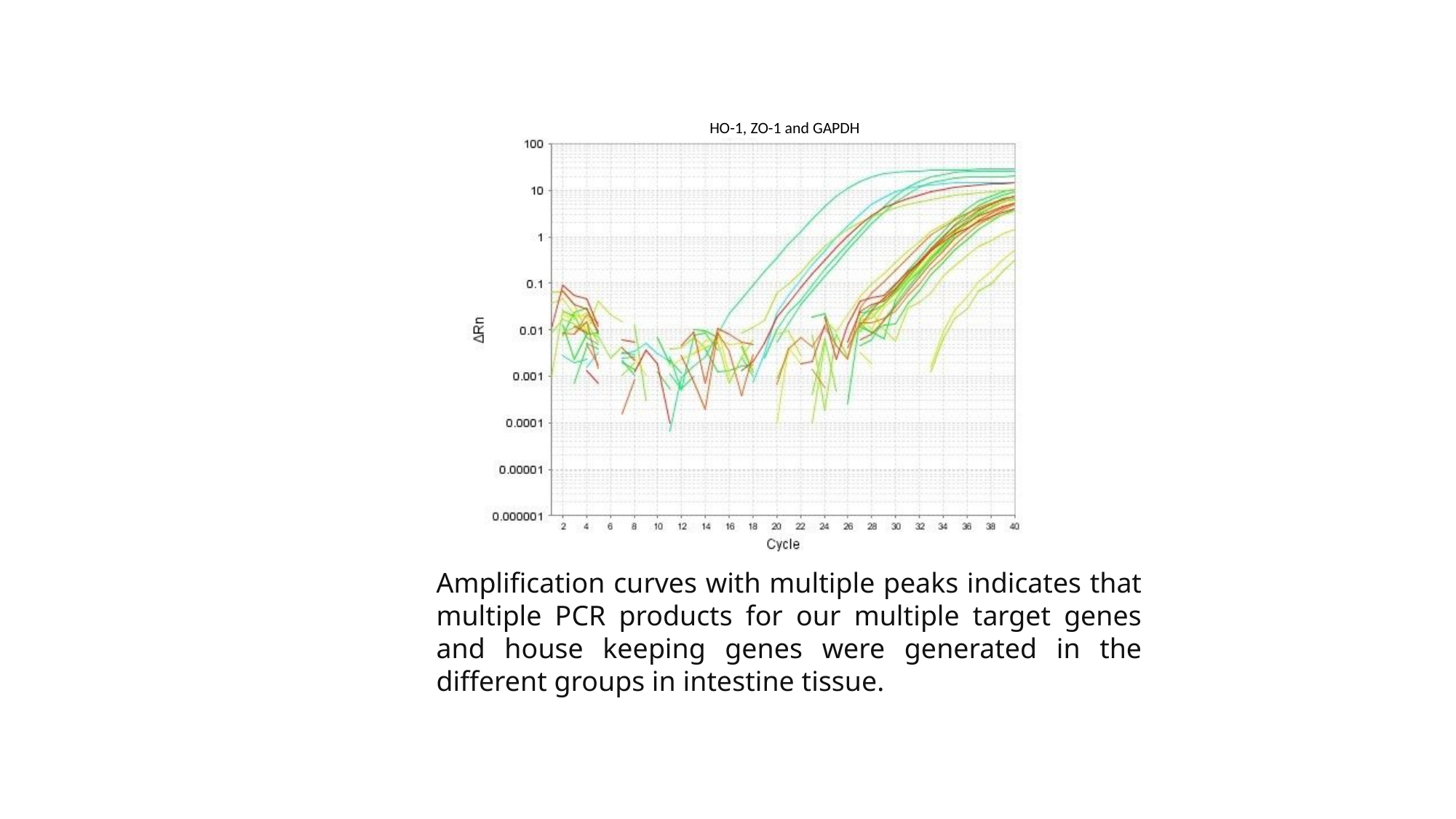

HO-1, ZO-1 and GAPDH
Amplification curves with multiple peaks indicates that multiple PCR products for our multiple target genes and house keeping genes were generated in the different groups in intestine tissue.
